# Linguistic Barriers in U.S. Neuropsychological Assessment: A State-of-the-Art Review

**DOI:** 10.1093/arclin/acaf091

**Published:** 2026-03-30

**Authors:** Ashby Martin

**Affiliations:** Department of Neurology, University of Iowa, Iowa City, IA 52242-1001, USA

**Keywords:** Interpreters, Neuropsychological assessment, Language proficiency, Cross-cultural test adaption, Literature review, Psychometrics

## Abstract

In the USA, 67.86 million people speak a language other than English at home. Many of these homes speak both English and a non-English language, with varying degrees of English fluency. A sizable 25.70 million people (8% of the U.S. population) report speaking, reading, or writing English “less than well” or “not at all.” These individuals are referred to as limited English proficiency (LEP) speakers. Such persons often need speech interpreters in health care and human service settings. When a speech interpreter is utilized to assess a LEP patient, the typical dyadic clinician–patient interaction shifts to a triadic clinician–interpreter–patient interaction. Although triadic interpreter-mediated interactions are commonplace in neuropsychological assessment when LEP patients are being evaluated, few guidelines exist for these interactions. To illustrate the current state of interpreter-mediated neuropsychological assessment in the USA, literature on the following themes have been reviewed: (1) existing language accessibility options; (2) the history of speech interpretation, clinically; (3) key distinctions in the profile of U.S. patients; (4) what linguistics barriers exist, have been addressed, and have arisen from the implementation of language accommodations in clinical practice; and (5) what recommendations have been established for the neuropsychological assessment of LEP patients. By detailing these five areas in the context of local, state, and national-level variability, this review identifies some of the systemic roots of linguistic barriers in the USA. Updated recommendation guidelines are outlined to promote efficacious and equitable neuropsychological assessment of LEP patients for implementation in clinical practice.

## INTRODUCTION

In the USA, 25.70 million people (8% of the U.S. population) report speaking, reading, or writing English “less than well” or “not at all.” These individuals are referred to as limited English proficiency (LEP) speakers. When clinical neuropsychologists assess LEP individuals, language accommodations are essential for efficacious and equitable encounters. Most often, an in-person professional medical speech interpreter is utilized (Nielsen et al., 2024), but language accessibility options vary widely across the USA and are often dependent on resource availability. Generally, “the lack of adequate interpreters and appropriate language services” is reported as the major barrier to LEP neuropsychological assessment (Fujii et al., 2022). In a national survey, clinical neuropsychologists reported “the lack of neuropsychological tests with appropriate U.S. norms for Hispanics/Latinos” and “the lack of neuropsychological instruments in Spanish” as the two most pressing barriers (Arango-Lasprilla et al., 2022). Referral sources, specifically outpatient neurology, report “time constraints” and “the lack of available face-to-face professional medical interpreters” as the largest barriers during LEP encounters (Tran et al., 2019). Understanding the nature of these barriers is a necessary step toward improving the validity and equity of neuropsychological assessment for LEP populations.

## METHODOLOGY

**Fig. 1:**
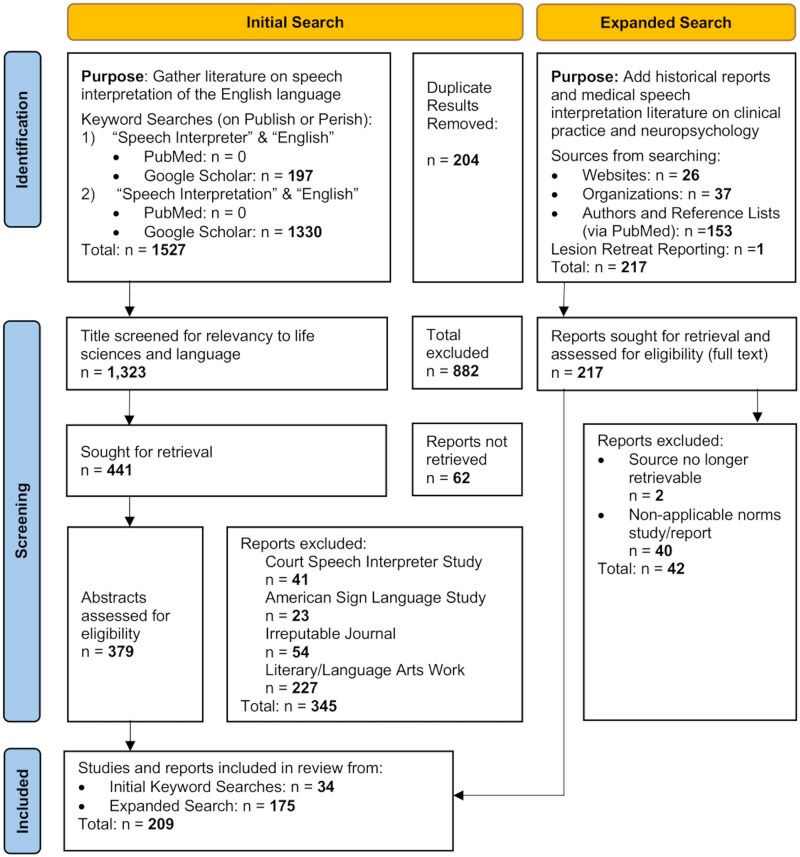
State-of-the-art-review methodology flow diagram. A multi-step search strategy yielded 209 sources for this state-of-the-art review. The PRISMA 2020 flow diagram (Page et al., 2021) was adapted to illustrate the methodological process. After sources were identified through keyword searches, they were screened for relevancy and reliability. The initial search is shown in detail, whereas subsequent searches—incorporating additional keyword queries, author-specific searches, and professional and governmental statistics—are summarized collectively under the expanded search portion of the diagram.

This review adopts a state-of-the-art (SotA) methodology to examine such language-related barriers in clinical neuropsychology in the USA. Specifically, it focuses on linguistic barriers faced by adult patients with LEP, although other age groups are mentioned when relevant. SotA reviews typically address (1) contemporary thinking about a topic; (2) historical progressions and patterns in the literature; (3) how modern perspectives have evolved over time; and (4) what direction the field could take moving forward (Barry et al., 2022). The overarching aim is to comprehensively detail each core area while promoting future inquiry on the topic. Typically, SotA reviews are variable in how sources are compiled and favor the concept that “there is no single objectively true or correct synthesis of a body of literature” (Barry et al., 2022). This flexibility makes SotA reviews especially suitable for topics that intersect multiple disciplines and, in the case of this review, well suited for a topic that has historical progressions and contextual detail sometimes tangential to the typical focus of clinical neuropsychology. Accordingly, although this review adheres to the general 3–4-pronged structure of a SotA review, it also incorporates a custom organizational framework intended to offer a degree of replicability, partly modeled after elements of systematic review methodology (see Fig.  1).

The review is organized into five sections, each aligned with one of the four aforementioned SotA categories, with one exception. The category *historical progressions and patterns in the literature* is addressed in two distinct sections: one focused on the clinical development of speech interpretation and the other on contextualizing key LEP populations relevant to assessment in the USA. To compile literature specific to each category, a multi-step search methodology was utilized. The initial search (see Fig.  1, left side) queried PubMed and Google Scholar using the academic tool *Publish or Perish* and the keywords “Speech Interpreter” AND “English” and “Speech Interpretation” AND “English.” This yielded 1,527 results, 204 of which were duplicates. The remaining 1,323 results were screened first by title and then by abstract. Results were excluded if the title explicitly referenced a legal matter, detailed a literature or language arts work, came from an irreputable journal, was specific to sign language, did not involve the English language, or was not conducted in the USA. Irreputable journals were defined as journals listed in databases of predatory publishers and journals, specifically journals listed on the updated Beall’s List (2024). This left 441 studies to review. After applying the established exclusion criteria to the full-text review, 34 studies remained. Because this initial search lacked specificity to clinical neuropsychology, a series of targeted follow-up searches were conducted and organized under the “expanded search” category (see Fig.  1, right side). Novel keywords such as “Clinical Neuropsychology,” “Interpreter,” “Assessment,” and “Limited English Proficiency” were incorporated and combined with prior keywords. These results were supplemented with statistics from governmental and professional associations, as well as author-specific searches when appropriate. This yielded an additional 175 sources, resulting in a final total of 209 sources included in the review. Whenever additional sources were incorporated, the expanded search boxes were updated accordingly to reflect the additions, as was the final tally of included sources.

## EXISTING LANGUAGE ACCESSIBILITY OPTIONS

### Language Accessibility Options in Hospitals and Clinics

Medical encounters, including neuropsychological assessments, rely on clear communication between clinicians and patients to proceed smoothly. Clear communication entails the correct exchange of information, allows for informed consent for treatment, and avoids breaches of patient–provider confidentiality (Avery, 1995). In order to facilitate clear communications between LEP patients and clinicians, hospitals and clinics in the USA generally provide several language accessibility accommodations that fall into three general categories: (1) in-person options, (2) remote options, and (3) supplemental options. Although the availability of certain language-accessible tools can be variable from hospital to hospital, a publicly available language accommodation is required by law to be available for all patients in the USA (Civil Rights Act of 1964, 1964; Office of Minority Health, 2013). Whether a clinician utilizes more than one language accessibility tool, however, is determined by a clinician’s selection or by the patient’s preference. Though patient preference is often secondary to clinician opinion and practical limitations during an initial visit (Karliner et al., 2007), patient preference can often dictate whether a service, or interpreter, is utilized in follow-up appointments (Flores, 2005).

Several in-person and remote options currently exist, including the typical triadic interpreter-mediated interactions with professional speech interpreters; low-to-no-cost options like community language banks in certain hospitals; remote options like telephonic, over-the-phone human speech interpretation and video-assisted speech interpretation; and even dyadic interactions with linguistically matching clinical staff as multilingualism grows in the USA (Fujii et al., 2022; Hasnain et al., 2006). Notably, only 4% of interpreter-mediated medical encounters utilized uncertified ad hoc interpreters, according to a recent study (Lion et al., 2021). This stands in stark contrast to prior decades when ad hoc interpreters were the most used language accessibility option (Putsch, 1985). Now, in-person and remote options are standard and are sometimes paired with additional supplemental options during neuropsychological assessment. For example, computer-assisted real-time transcription (CART) is a semi-automated, real-time speech-to-text captioning service that can support patients with hearing difficulties during assessment, either alongside a speech interpreter or as a complement to another language-accessible option. For additional details on all the mentioned language accessibility options, and several more options not mentioned, see Table 1. Table 1 also provides insight into whether each approach is (1) dyadic, (2) triadic, or (3) variable in structure, as well as the specific category—in-person, remote, or supplemental—each language accessibility option falls into.

**Table 1:** Language accessibility options offered in hospitals or clinics for neuropsychological assessment

**Language accommodation**	**Description**	**Interaction structure**	**How service is typically rendered**
Linguistically matching clinical staff	An American Board of Clinical Neuropsychology (ABCN)/American Board of Professional Psychology (ABPP) board-certified clinical neuropsychologist fluent in the same non-English language as the patient. They directly administer the assessment	Dyadic	In-person
Linguistically matching non-clinical staff	An assisting staff member (such as a technician, psychometrist, post-doctoral fellow, or other non-ABCN/ABPP-certified staff) who work under the direct supervision of the ABCN/ABPP board-certified clinical neuropsychologist, fluent in the same non-English language as the patient. They directly administer the assessment under supervision	Triadic	In-person
Professional speech interpreters	An individual who has obtained professional licensure (such as a CCHI or a NBCMI license) in medical interpreting. These individuals are fluent in the same non-English language as the patient and can assist the clinical neuropsychologist in administering the assessment	Triadic	In-person
Community language banks	These structured networks are public service organizations that connect individuals to trained volunteer interpreters or translators. Professional licensure (such as a CCHI or a NBCMI license) is not certain, but service is typically free, or low-cost and funded by nonprofit or community-driven initiatives. These individuals are typically fluent in the same non-English language as the patient and would assist the clinical neuropsychologist in administering the assessment.	Triadic	In-person
Ad hoc interpreters	This classification typically denotes individuals who are uncertified, untrained volunteers offering language aid and includes language brokers. Typically, these are collaterals of the patient but can describe any unqualified individual. Although typically fluent in the same non-English language as the patient, this last-resort aid would attempt to assist the clinical neuropsychologist administer the assessment	Triadic	In-person
Collateral-mediated verbal input	This term can be used to describe instances during the patient interview or feedback session, where the collateral (such as family and friends) provides input on behalf of the patient. These individuals are typically fluent in the same non-English language as the patient. They do not assist the clinical neuropsychologist in administering the test battery	Variable: triadic or larger	In-person
Over-the-phone human speech interpretation services	An individual who has obtained professional licensure (such as a CCHI or a NBCMI license) in medical interpreting and is not physically present at the hospital or clinic room, aids in the assessment. These individuals are fluent in the same non-English language as the patient, and they assist the clinical neuropsychologist in administering the assessment over-the-phone	Triadic	Remote
Fully automated telephonic speech translation services	A non-human operated call number or artificial intelligence service that provides language services (such as Google Translate or ChatGPT). These services typically have no professional licensure qualifications in speech translation and are not typically utilized in administering the assessment. Patients may utilize these services at times during the patient interview or feedback session	Dyadic	Remote
Semi-automated telephonic speech interpretation services	These hybrid services utilize human interpreters accessed through automated technological platforms for remote interpretation assistance. These platforms (such as CyraCom) utilize interpreters who have obtained professional licensure (such as a CCHI or a NBCMI license) in medical interpreting. These individuals are fluent in the same non-English language as the patient and can assist the clinical neuropsychologist in administering the assessment through their respective technology platform over-the-phone	Triadic	Remote
Video-assisted human speech interpretation services	These hybrid services utilize human interpreters accessed through technological platforms for remote interpretation assistance. These platforms (such as CyraCom or GLOBO) utilize interpreters who have obtained professional licensure (such as a CCHI or a NBCMI license) in medical interpreting. These individuals are fluent in the same non-English language as the patient and can assist the clinical neuropsychologist in administering the assessment through their respective technology platform via remote video-assisted services	Variable: dyadic or triadic	Remote
Shadow interpreters or stand-by interpreters	These individuals have obtained professional licensure (such as a CCHI or a NBCMI license) in medical interpreting and are fluent in the same non-English language as the patient. They act in a secondary or non-participatory role to the clinical neuropsychologist during assessment. They primarily serve to aid in situations where a patient refuses interpreter-mediated assessment or in situation where the clinician’s fluency in the same non-English language as the patient is questionable. During assessment, they document omissions and inaccuracies that occurred, and they may intervene with corrections during these instances	Triadic	Supplemental
Translation-centric	This approach refers to nonverbal communication utilizing written, or typed, language. Although automated services (such as Google Translate or ChatGPT) fall under this approach as well, translation-centric approaches also include prewritten and in-person translations provided by individuals who have obtained professional licensure (such as a CCHI or a NBCMI license) in medical interpreting. These methods can assist in situations of pronunciation difficulties or heavily accented speech during assessment	Variable: dyadic or triadic	Supplemental
Simultaneous-consecutive profession interpretation (SimConSec)	A notable variation of typical interpretation practices, rarely utilized in medical settings, where an individual who has obtained professional licensure (such as a CCHI or a NBCMI license) in medical interpreting utilizes a mix of simultaneous and consecutive speech interpretation through the aid of supplemental techniques or tools. These individuals are fluent in the same non-English language as the patient and can assist the clinical neuropsychologist in administering the assessment	Triadic	Supplemental
Computer-assisted real-time transcription (CART)	A form of real-time transcription where spoken words are converted into written text by a specialized machine (such as stenography equipment). Spoken words appear as text on a screen, device, or online and can be the same language, or a translation to another language. CART is often utilized in conjunction with a professional speech interpreter, or with a linguistically matched clinician, to assist the clinical neuropsychologist in administering the assessment to patients who are hard of hearing	Variable: dyadic or triadic	Supplemental

*Note:* Outlined are language accessibility options offered in clinical settings for neuropsychological assessment. The columns specify the option; provide a brief description; indicate whether the interaction is dyadic, triadic, or variable; and describe how the service is typically rendered (in-person or remote). When an option is commonly utilized in conjunction with another, it is listed as supplemental.

However, despite the growing pool of language-accessible tools, not every clinician utilizes all available options, nor do they value these tools equally. For example, in a survey of 861 U.S. hospitals, it was reported that although telephonic interpretation services were the most widely available option, at 92% availability, they were less frequently utilized by clinicians than in-person speech interpreter options, despite in-person speech interpreters only being an option in 68% of hospitals (Hasnain-Wynia et al., 2006). This suggests a preference for direct interaction despite broader access to remote services.

### Vocal and Nonvocal Communication Variability

**Fig. 2:**
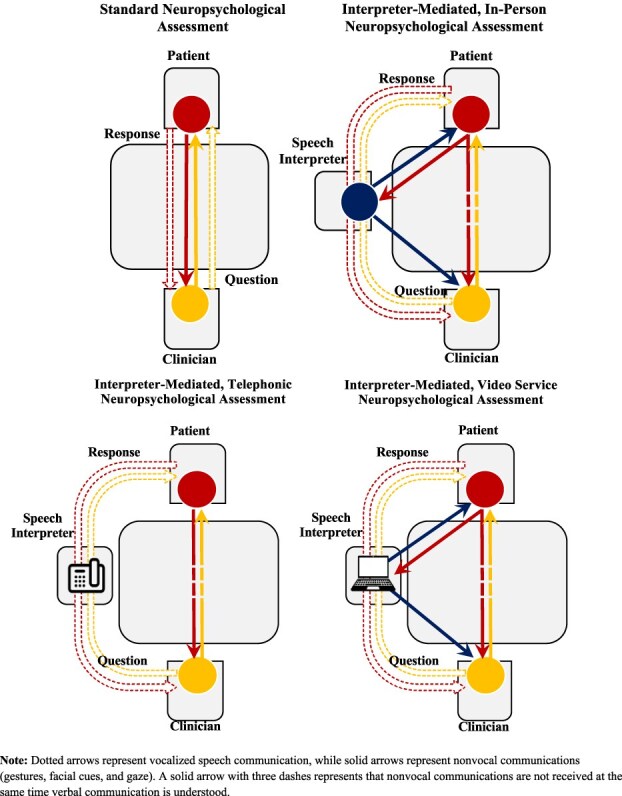
Vocal and nonvocal communication variability during neuropsychological assessment. Four forms of neuropsychological assessment are depicted: Three triadic interpreter-mediated formats (in-person, telephonic, and video service) and the standard dyadic format. Directionality and delays in communication are depicted using dotted and solid arrows: dotted arrows represent vocalized speech communication, solid arrows represent nonvocal communications (gestures, facial expressions, gaze), and a solid arrow with three dashes represents that nonvocal communications are not received at the same time verbal communication is understood.

Although no experimental studies have directly tested whether telephonic speech interpretation affects cognitively demanding tasks like neuropsychological assessment, qualitative research highlights several concerns (Gracia-García, 2002; Hadziabdic et al., 2011; Lion et al., 2015; Price et al., 2012; Schulz et al., 2015). Telephonic interpretation eliminates nonverbal cues—such as gestures, facial expressions, and eye contact—which are critical for accurate clinician–patient communication during assessment (Kelly, 2008; Schulz et al., 2015). These limitations, combined with verbal delays and the absence of visual alignment between interpreters and patients, suggest that telephonic services may hinder communication quality in neuropsychological contexts (see Fig.  2).

Thus, as telephonic services became more widespread in the late 2000s, speculation on potential adverse effects became more common (Gracia-García, 2002; Kelly, 2008). When 53 medical speech interpreters in the USA were surveyed on the efficaciousness of clinical encounters, telephonic interpretation was unilaterally rated worse than video-assisted human speech interpretation services for psychosocial scenarios (Price et al., 2012). Telephonic interpretation was rated worse in case management, hospital discharge, consent, therapy encounters, when debriefing patients, and when debriefing collaterals like family members. Notably, only 25% of respondents felt collaterals were adequately debriefed when telephonic services were utilized (Price et al., 2012). Similarly, a survey of 208 LEP parents found that comprehension of their child’s diagnosis was significantly worse with telephonic interpretation (59.8%) than with video-assisted interpretation (74.6%) (Lion et al., 2015). International literature reviews also suggest a preference for video-service interpretation over telephonic interpretation; however, none recommend against the use of telephonic interpretation for neuropsychological assessment (Franzen et al., 2021; Joseph et al., 2017). Specifically, current field recommendations advocate for the use of other language accessibility options when available, rather than recommending against telephonic interpretation (Franzen et al., 2021; Nielsen et al., 2024). Moreover, with the growing adoption of multimodal audiovisual tools in hospitals, this gap in the literature may ultimately be addressed through technological shifts, potentially reducing the urgency for future experimental study.

### Artificial Intelligence in Speech Interpretation

Artificial intelligence (A.I.)–driven speech interpretation is increasingly prominent in discussions of medical interpreter services, reflecting a broader technological shift in healthcare (Genovese et al., 2024; Taira et al., 2021). Although several studies have attempted to replicate aspects of human interpretive services, most demonstrated limited effectiveness and are now dated relative to recent advances in A.I. (Aiken et al., 2010; Elbayad et al., 2020; Hamon et al., 2009; Odoje, 2015; Paulik & Waibel, 2009; Sangeetha & Jothilakshmi, 2017; Sridhar et al., 2013). At present, A.I. is regarded as a supplemental tool in clinical contexts rather than a replacement. It remains less effective than human interpreters, particularly for context-sensitive communications (Genovese et al., 2024; Taira et al., 2021). This limitation is especially apparent in neuropsychological assessments. When A.I. was used to administer assessments, it was concluded that “conducting a cognitive assessment needs to be administered by a trained professional, which is costly and time consuming” (Weaver et al., 2013). A.I. should not be considered a substitute for a neuropsychologist, though it may have supplementary applications as the technology continues to evolve.

A key factor driving these attempts may be the financial burden U.S. hospitals face in providing interpreter services. Unlike most U.S. healthcare costs, which are typically billed to patients or insurance providers, the full cost of speech interpreter services is legally mandated to be covered by the business or government entity, not by the individual LEP patient receiving the services (U.S. Department of Justice [DOJ], 2010; Office of Minority Health, 2013). These mandates have been in place since 2000, when the U.S. Department of Health and Human Services introduced the National Standards for Culturally and Linguistically Appropriate Services in Health Care (CLAS Standards) to support compliance with Title VI of the Civil Rights Act of 1964 (Office of Minority Health, 2001). CLAS was expanded in 2013 to expand clinical standards to institutions and is still in effect today (Office of Minority Health, 2013). As a result, hospitals bear the expense of providing interpreter services 97% of the time (Hasnain-Wynia et al., 2006). This financial imbalance creates a strong incentive for hospitals to explore cost-saving alternatives such as automation, despite limited effectiveness in the past (Genovese et al., 2024). Overall, automation represents just one of many options available to LEP patients for critical medical encounters, like neuropsychological assessments. However, to fully understand the broader context of speech interpretation in clinical neuropsychology, it is important to consider the historical development of medical speech interpretation. Although the modern landscape of medical speech interpretation has been established to some extent in the current section, the historical foundations that shape it are not often discussed or thoroughly reviewed in existing literature.

## THE HISTORY OF SPEECH INTERPRETATION CLINICALLY

### The Field of Community Speech Interpretation

Although speech interpreters were used as early as the third millennium b.c. in ancient Egypt (Mikkelson & Jourdenais, 2015), the formal study of speech interpretation and its role in medical contexts emerged only relatively recently. A seminal work on the subject, *Introducing Interpreting Studies* (Pöchhacker, 2003), was published by Routledge in 2003. Earlier efforts to establish the field of Community Speech Interpretation appeared in the previous decade, with Cecilia Wadensjö widely recognized as a foundational figure in interpreter-mediated medical encounters (Wadensjö, 1992). Wadensjö adapted concepts developed for effective educational practice—such as dialogic discourse in triadic interactions—and applied them to medical settings, framing speech interpretation as a dynamic, non-normative, and dialogical process. Wadensjö sought to address critical language barriers that clinicians faced in medical environments. At the time, language barriers were so severe that clinicians reported instances where neither the patient nor their collaterals (e.g., family members) were informed of the patient’s condition due to concerns that their clinical judgments would be misinterpreted—even when the information involved vital end-of-life issues (Thornton et al., 2009).

Although Wadensjö and other early pioneers in the field shared their research in professional settings, the dissemination of their insights remained limited. The *Critical Link: Interpreters in the Community* conferences provided a rare early forum for discussion and collaboration, but the knowledge shared largely remained within these conference settings rather than being widely accessible to the broader medical or interpreting communities (Carr et al., 1997). The lack of a structured framework meant that advancements in the field were often siloed, preventing the development of standardized practices. This changed with the publication of Pöchhacker’s, 2003 seminal work, which synthesized previous research and discussions into a structured framework that laid the foundation for the modern study of Community Speech Interpretation (Pöchhacker, 2003). The medically specific follow-up work, *Healthcare Interpreting: Discourse and Interaction*, in 2007, further refined these standards within the healthcare context (Pöchhacker & Shlesinger, 2007). Prior to such structure, literature on speech interpreter practices was sparse, inconsistent in terminology, and highly variable when defining credentialing and certification standards (Carr et al., 1997).

Vocational training programs for interpreters reflected this lack of standardization. The first integrated curriculum in the USA for the training of speech interpreters was established in 1967 at the Monterey Institute of Foreign Studies in Monterey, California (Arjona, 1978). It set the first institutional standards for language interpreter education in the USA, but its curriculum was designed for general vocational and military applications rather than as a specialized training program for healthcare implementation. Professional medical interpreter programs were not standardized in clinical practice until years later. The University of Iowa Hospitals and Clinics (UIHC) established its Interpreters and Translation Program in 1974, specifically to serve healthcare patients. As a seminal social service program, it was directly integrated into Iowa’s largest and most utilized hospital to ensure language interpreters were available to both LEP and deaf patients. UIHC’s program pioneered structured models of hospital-based speech interpretation in the USA (Díaz-Duque, 1982).

Despite such institutional advancements, no formalized state or national certification system existed to ensure a consistent standard of practice beyond the local level for years. Only after multiple lawsuits did the first state-level certification process begin as a pilot program in Washington State in 1993, becoming formally established at the start of 1995 (Woloshin et al., 1995; Youdelman, 2013). Shortly after, the Massachusetts Medical Interpreters Association (MMIA) introduced guidelines for medical interpreters in October 1995, providing a template for other states to develop certification practices (Avery, 1995). Similarly, the establishment of the CLAS Standards in 2000 gave hospitals and health care facilities a blueprint for basic communication and language standards for LEP patients in need of language assistance (Office of Minority Health, 2001). Nationally, these standards established speech interpreters and other language accessibility options as essential services that must be provided free of additional charge, recognized language as multimodal, and discouraged the use of ad hoc interpretation (Office of Minority Health, 2001). They also promoted certification processes, despite no national certification systems for medical speech interpreters existing at the time. It was not until late 2009, with the launch of the Certification Commission for Healthcare Interpreters (CCHI) in September, that a national certification process came into existence (Youdelman, 2013). However, once one did, the next soon followed: within a month, the National Board of Certification for Medical Interpreters (NBCMI) released their certification program in October (Arocha & Joyce, 2013).

Unfortunately, both state and national certifications remain voluntary, with no legal requirement for interpreters to obtain them before practicing professionally. This lack of regulatory oversight persists even amid modern efforts to standardize the profession. In 2024, Washington attempted to mandate professional licensing for employed interpreters, but the legislation ultimately failed in engrossment (Washington State Senate, 2024). Although the field of medical speech interpretation remains relatively young, calls for formalized training and regulation in the USA date back as early as 1951 (Andersen, 1951), underscoring the longstanding recognition of its necessity.

### Summary Domains of Speech Interpretation and Language Translation

When MMIA standards were set in 1995, existing terminology became formally defined and categorized for the first time. Since then, several updates have been added, and three summary domains can be utilized to describe the major components of speech interpretation: (1) interactional variability, (2) the mode of interpretation, and (3) the setting where interpretation occurs (Agrifoglio, 2004; Blackstone et al., 2015; Gile et al., 2010; Mikkelson & Jourdenais, 2015; Pöchhacker, 2003; Pöchhacker & Shlesinger, 2007; Rivera Mindt et al., 2008; Viezzi, 2013). The first domain, interactional variability, details the form and variety of the utterance of a word during triadic interactions and can be further defined by subcategories like speed and intonation, accompanied gestures, dialect, slang and figures of speech, legal and medical jargon, and lisp and stutter. The second domain, the modality, is in reference to the form of delivery speech interpretation occurs. This includes defining whether the interpreter utilizes orally spoken or visually signed communication; the mode of delivery (e.g., consecutive or simultaneous interpreting); the directionality between a patient, clinician, and interpreter speech; and the use of technology or machine interfaces. The third domain, setting, refers to location, standards of cleanliness and upkeep, time of day, visitation frequency and recency of incident, and noisiness of the room. The setting also involves considerations that relate to the current political climate, historical context, cultural variability, and socioeconomic circumstances.

Across these three domains, select terminology warrants explicit mention and definition for later application purposes. First and foremost, interpreter-mediated medical encounters should not be defined merely as “oral translation” of instruction and response. As outlined in *Healthcare Interpreting: Discourse and Interaction,* Pöchhacker defines interpreting as translational activity that is distinguishable in temporal immediacy and encompasses signed, as well as spoken languages, with respect to the localized medical register (Pöchhacker & Shlesinger, 2007). Pöchhacker asserts that interpretation of speech draws on eight components: (1) medium, (2) physical setting, (3) mode (oral or visual), (4) languages (cultures), (5) discourse (register), (6) participants (education/age), (7) interpreter qualifications, and (8) problem (reason for neuropsychological assessment). All eight components can individually vary from one assessment to another, and each bears some individual consideration when critiquing speech interpreter-mediated assessment compared to non-assisted clinician assessment.

### Speech Interpreter and Language Translation Terms and Definitions

Several key terms within the domain of interactional variability merit description. *Register* refers to the social or intellectual level at which language is used (Díaz-Duque, 1982). All languages feature multiple registers, and neuropsychological assessments require a specific standard of register to ensure comparable accuracy across assessments (Mikkelson & Jourdenais, 2015). Misinterpretation and oversimplification often occur when assessments are interpreted by non-medical professional interpreters (Lezak et al., 2012; Rivera Mindt et al., 2008). When examining this issue, it is critical to assess whether the speech interpreter has identified the LEP patient’s individual language register and whether the information they are providing aligns with that register. When the LEP patient’s register is not considered, *nodding syndrome* can occur, where a patient nods in agreement despite not understanding information, often out of fear of embarrassment (Mikkelson & Jourdenais, 2015). Nodding syndrome is more common with less experienced interpreters or those who simplify the assessment register beyond acceptable limits (Van Besien & Meuleman, 2008).

Although simplifying terminology into everyday language may feel logical, it more often negatively affects assessment quality. For example, speech interpreters at UIHC are explicitly instructed to not “change the register (i.e. turn technical language into everyday language)” to maintain a strict assessment standard (GLOBO, 2024, p. 4). Specifically, poor word choice has been strongly linked to lower standards accuracy and reliability of assessment tools (Van Besien & Meuleman, 2008). Due to the polysemic nature of word meaning, substituting words can readily change what a question is asking without anyone necessarily being aware of the change (Herman & Saputra, 2002; Van Besien & Meuleman, 2008). Additionally, the *right context effect* can negatively affect the quality of speech interpretation during assessment. This effect is specific to cases of consecutive speech interpretation and occurs when free recall from an interpreter significantly overrepresents content from the end of a speech segment in interpreter speech (Dahan, 2010).

*Consecutive interpretation* (CI) is one of two prominent modalities of speech interpretation alongside *simultaneous interpretation* (SI). Both characterize the summary domain of mode in professional speech interpretation, and each has several relevant subcategorizations (Agrifoglio, 2004; Avery, 1995; Pöchhacker & Shlesinger, 2007). SI involves real-time spoken speech interpretation, typically overlapping with the speech of the initial speaker, whereas in CI, the interpreter waits for the speaker to pause at points before rendering interpretation to the patient or clinician (Pöchhacker, 2016). Typically, longer delays between pauses are facilitated by physical note taking during CI, referred to as “classical” CI, whereas short bursts without additional items are referred to as “short” CI, or *liaison interpreting*. Notable SI subcategories include whisper/*chuchotage* for small group SI, *respeaking*/speech-to-text where subtitles appear as words are being spoken, and *sight translation* which involves verbally translating written word. Sight translation most often occurs when written materials are not provided in the desired language and an interpreter must verbally translate the text to the listener, as is the case when language-specific neuropsychological assessments are not yet transcribed into new languages. SI and CI can cooccur as well, as is the case with the use of *SimConsec* (see Table 1 for description of SimConsec as a language accessibility option) (Pöchhacker, 2016).

### Non-professional Speech Interpretation

Other, non-professional modes of speech interpretation also exist. *Ad hoc interpreters* are non-credentialed multilingual friends, family members, or spouses that provide language support to the LEP patient but lack the requisite register of medical speech (Bührig & Meyer, 2006; Woloshin et al., 1995). A common subtype is *language brokering*, in which children interpret for their LEP family members, often in power-imbalanced situations due to notable age gaps (Iqbal & Crafter, 2022). Prior to the implementation of certification standards, such as the MMIA guidelines introduced in 1995, ad hoc interpreters were the most common form of language support for LEP patients (Putsch, 1985; Woloshin, 1995). At that time, only 15% of medical encounters involved professional interpreters, 39% relied on ad hoc interpreters, and 46% provided no language assistance at all (Baker et al., 1996). This pattern persisted regionally into the early 2010s, with up to 50% of interpreted encounters using ad hoc methods (Flores et al., 2012; Ginde et al., 2009). However, usage has since declined as the adverse effects of ad hoc interpretation became more widely recognized (Flores, 2005).

Ad hoc interpreters have been linked to numerous issues, including inadequate communication of medication side effects (David & Rhee, 1998), reduced patient satisfaction (David & Rhee, 1998; Kuo & Fagan, 1999; Lee et al., 2002), and frequent interpretation errors, such as omissions, distortions, redundancies, and irrelevant information (Ebden et al., 1988; Flores et al., 2003; Launer, 1978). In neuropsychological contexts, such errors could result in misidentification of symptoms, normalization of pathological behaviors, and misinterpretation of affect and thought processes (Marcos, 1979). At UIHC, ad hoc interpreters were reported to violate confidentiality, omit diagnostic details, and generalize or speculate in their summaries, contributing to inaccurate or incomplete assessments (Díaz-Duque, 1982). For these reasons, ad hoc interpretation is strongly discouraged in neuropsychological assessment (Fujii et al., 2022; Nielsen et al., 2024). As a result, ad hoc interpretation usage is as low as 4% in Seattle Children’s Hospital (Lion et al., 2021).

Professional interpreters outperform ad hoc interpreters in accuracy, comprehension, outcomes, and satisfaction (Karliner et al., 2007). Although both professional interpreters and ad hoc interpreters make errors in their interpretations, the errors made by ad hoc interpreters are more likely to have potential clinical consequences (i.e., 77%) than those committed by professional, hospital interpreters (i.e., 53%; Flores et al., 2003). Errors by professional interpreters included omissions of allergy-related questions, incomplete instructions for medication, and miscommunication of patient rights. These errors are rare when language assistance is not required and may be less frequent with language-concordant clinicians (Seijo et al., 1991). In neuropsychology, bilingual clinicians often avoid using interpreters entirely, which may reduce risks associated with interpretation error (Arango-Lasprilla et al., 2022). Although further research is needed, the existing literature makes clear that interpreter-mediated assessments are prone to significant communication errors, with ad hoc interpreters introducing the greatest risk.

### Masking Effects during Speech

Only one empirical study, conducted in Spain, found ad hoc interpreters performed as well as professional interpreters (Cooke et al., 2008). This study specifically introduced the cocktail party problem—background distraction noise—and supplemental co-speech gesturing during SI medical encounters to produce this effect (Arbona et al., 2024; Cooke et al., 2008). Situations like the cocktail problem specifically manipulate aspects relevant to the domain of speech interpreter setting. The cocktail problem involves two different types of masking possible during conversation: *energetic masking* and *informational masking*. Energetic masking refers to distractional noise that partially masks the verbalized content spoken by a speaker, whereas informational masking refers to distractional noise loud enough that one cannot fully hear content verbalized by an individual (Oviatt, 2000). In closed rooms, masking effects become increasingly problematic as the number of speakers increase (Cooke et al., 2008). Although dyadic assessments between a clinician and a patient would be unlikely to be hindered by masking effects, triadic assessments—with a clinician, an LEP patient, and a medical speech interpreter present—can be significantly hindered. If the patient is a foreign-born LEP individual, informational masking can be even more detrimental. Non-native Spanish/English LEP patients reportedly perform 10%–30% worse on listening tasks than native listeners due to informational masking (Cooke et al., 2008; Oviatt, 2000). One explanation, posited by the *Communications of the ACM* speech textbook (2000), suggests that English-familiar speakers are better able to discern spoken phonemes from visible lip movements in noisy environments compared to non-native LEP speakers (Oviatt, 2000). Regardless of the reason behind masking effects, they are largely avoidable during interpreter-mediated neuropsychological assessments with proper turn-taking protocols, though they can remain a concern in uncontrollable noise-compromised situations—such as construction noise or sound generated by medical equipment.

### Shadow Interpreters

Other precautions can also be implemented to support LEP patients during assessment. In O’ahu, Hawai’i, *shadow interpreters* have been utilized to assist LEP patients during presurgical and postoperative consultations (Kostareva et al., 2023). Originally coined in a legal setting as *stand-by interpreters* (Angermeyer, 2008, p. 390), the term has recently been adapted to shadow interpreters in hospital settings due to its parallels with clinical shadowing. A shadow interpreter is a professional interpreter who takes on a secondary, non-participatory role, typically when a patient declines interpreter-mediated communication or when the clinician’s fluency in the patient’s language is uncertain. In such cases, the clinician communicates directly with the patient while the shadow interpreter documents linguistic errors or intervenes if clarification is needed. This approach may also benefit bilingual clinical neuropsychologists who are linguistic matches but not dialectic matches to an LEP patient during assessment. Though this application has yet to be formally explored, the use of such precaution can help mitigate indirect cultural factors that have been shown to significantly influence outcomes. Specifically, cultural factors have been reported to inadvertently reframe the context of speech interpretation and confound the quality and accuracy of neuropsychological assessments (Casas et al., 2012; Li, 1999). Thus, further exploring the cultural complexities faced by LEP patients may help explain why professional medical interpreters have higher error rates in some hospitals but not others and why misinterpretation of test instructions remains a significant challenge in neuropsychological assessment.

### Cultural Variability in LEP Patients and Cognitive Bias

Cultural variability is robustly supported as one of, if not the largest, factor to consider when discussing avenues for improvement in neuropsychological assessment (Ardila, 2005; Brickman et al., 2006; Fernández & Marcopulos, 2019; Lezak et al., 2012; Rivera Mindt et al., 2010). However, despite its recognized importance within neuropsychology, cultural variability remains one of the least quantifiable and least systematically addressed variables in patient–clinician interactions (Echemendia et al., 1997; Fernández & Marcopulos, 2019; Howieson, 2019; Richards et al., 2015). This disconnect may be partially attributed to cognitive biases—particularly *extension neglect*—which can obscure the perceived significance of culture in clinical decision-making (Loewenstein et al., 1994; Miles, 2013; Richards et al., 2015). Extension neglect occurs when the scale of an issue or finding is not properly taken into consideration, leading individuals to assign equal importance to issues of vastly different scope (Kahneman, 2000). In cases of extension neglect, an individual assigns the same weight to a small-scale issue as they do to a large-scale issue, and both issues are addressed with equal importance rather than in proportion to the true scale of the issue. This often leads to underestimating or overestimating risks or benefits due to a failure to consider overall proportions and can affect both the norming and interpretation of neuropsychological assessment. Similarly, the application of assessment norms can suffer from extension neglect when norms are generalized to all groups or are utilized too narrowly by clinicians (Nead et al., 2022).

#### Extension neglect in literature

In a similar vein, extension neglect can negatively affect one’s interpretation of LEP patient statistics. Percent values, such as the statistic that 8% of people are LEP (U.S. Census Bureau, 2021), can obscure the true scale of the phenomena. There are 25.7 million people in the USA who are not fluent in English (U.S. Census Bureau, 2021; Haldar et al., 2023). Similarly, linguistic and cultural diversity statistics tend to be misconstrued due to extension neglect. For instance, although it is statistically true to say that the USA has a significantly lower proportion of multilinguals than the average proportion in Europe—an estimated 21.66% in the USA versus an estimated 74.6% in Europe (U.S. Census Bureau, 2022; Eurostat., 2022)—such a comparison does not fully capture the scale of multilingualism in the USA. There are 67.86 million people in the USA who report speaking a language other than English at home. To reduce extension neglect, percentages should be presented alongside raw numbers, especially when evaluating disparities in healthcare. This is particularly important in clinical neuropsychology, where concerns about interpreter use can be misjudged as being menial. Every patient, regardless of language status, deserves a standard of care that reflects the scale of their needs (American Psychological Association [APA], 2002; U.S. Department of Health and Human Services, 2020; U.S. Department of Justice, 2002). LEP status should not diminish or limit that expectation.

## KEY DISTINCTIONS IN THE PROFILE OF U.S. PATIENTS

### LEP Demographics in the USA

Contextualizing statistics is important. Multilingualism and the Hispanic/Latino population are growing rapidly in the USA, making LEP-related statistics quickly outdated (Crown et al., 2024). From 2022 to 2023 alone, Hispanics/Latinos accounted for 71% of total U.S. population growth, and Hispanic/Latino individuals now representing 1 in 5 Americans, or 65.2 million individuals (U.S. Census Bureau, 2024). The majority are of Mexican origin (63%), followed by Puerto Ricans (9%) and Cubans (4%) (U.S. Census Bureau, 2017). Hispanic/Latino individuals comprise the majority of LEP patients (Haldar et al., 2023), and Spanish is by far the most requested language for interpreter services in neuropsychology—used by 63% of LEP individuals (16.19 million), compared to just 7% (1.8 million) for Chinese languages (U.S. Census Bureau, 2021). However, high prevalence of Hispanic/Latino LEP patients and high prevalence of Spanish interpreter requests by LEP patients does not necessarily mean every Hispanic/Latino in the USA is requesting that a Spanish interpreter be present during a neuropsychological assessment. So, although not representative of every case, a specific focus on Hispanics/Latinos with Spanish considerations bears additional focus.

### Spanish Language in the USA

In the USA, Spanish language growth is rapid and Hispanic/Latinos represent the largest Spanish-English speaking group (U.S. Census Bureau, 2020). Nationally, ~42 million people speak Spanish, with Spanish comprising 61.6% of all non-English languages spoken (U.S. Census Bureau, 2022). Linguistic growth is particularly evident in education, where Spanish and other non-English languages are increasingly integrated into curricula rather than excluded (Alfaro & Bartolomé, 2023), marking a shift from prior decades (Barrett et al., 2022). Specifically, the number of students in the USA who grew up with two languages has grown by 24% over the last two decades (Rosenblum & Hipsman, 2016). As a result, although only 21.63% of the U.S. general population speaks more than one language (U.S. Census Bureau, 2020), among the children aged 8 and under, roughly one in three are reportedly multilingual (Park et al., 2018).

### Migrant Variability and Additional Variability in U.S. Hispanics/Latinos

Additional variability in language use and health outcomes exists between U.S.-born and immigrant Hispanics/Latinos. Although 75% of all Hispanics/Latinos report speaking Spanish, this rises to 93% among immigrants and falls to 57% among U.S.-born individuals (Pew Research Center, 2022). Similarly, although 28% of all Hispanics/Latinos report limited English proficiency (LEP), only 9% of U.S.-born individuals do, compared to 62% of the foreign-born population (Pew Research Center, 2023). Immigration status also influences healthcare access and experience. For example, Mexican immigrants report higher rates of discrimination in health settings than their U.S.-born counterparts (Viruell-Fuentes, 2007). Hispanics/Latinos are also at elevated risk for conditions like cardiovascular disease and diabetes, which are both linked to later-life cognitive decline (Elfassy et al., 2019; González et al., 2020; Lamar et al., 2019; Pérez-Stable et al., 1997). They also have the lowest national levels of health literacy, with LEP cited as the main barrier (Kutner et al., 2006; Office of Minority Health, 2023). Similarly, compared to U.S.-born LEP individuals, immigrant LEP Hispanics/Latinos are less likely to seek or receive care and less likely to schedule medical visits (DuBard & Gizlice, 2008; Shi et al., 2009). These patterns suggest that foreign-born LEP individuals undergoing neuropsychological assessment may face greater barriers to care and communication than what can be typically expected by merely LEP status.

Language use also varies by context. Spanish–English bilinguals in Massachusetts, for example, often switch languages based on setting, using Spanish at home and in shops, and English in formal environments like schools or hospitals, even when English is not the dominant language (Solorzano Jr., 2009). This flexible use of language, commonly referred to as *code-switching*, is observed across bilingual communities worldwide. In Iowa, similar context-dependent shifts have been documented, shaped by local social and cultural norms (Bakos et al., 2010). In England, Mann and de Bruin (2021) identified nine distinct contextual domains through which bilinguals adjust language use, and Rayo and coworkers (2024) found code-switching behaviors to be strongly influenced by sociocultural factors across several international contexts. As a result, cognitive frameworks have been developed to account for some of this variability (Tiv et al., 2022), and emerging neuropsychological norming efforts are beginning to reflect these contextual influences (Suárez et al., 2020). Whether national frameworks align with local trends, however, depends on how comparable those populations truly are, a point examined in the next section through a comparison of Iowa’s LEP demographics to national averages.

### LEP Demographics in Iowa Compared to National Averages

Overall, when considering multilingual ability, representation at the migrant level, representation at the student level, and across Spanish-speaking groups, Iowans consistently have less linguistic diversity than national U.S. averages but have higher rates of growth when compared to prior year estimates (Migration Policy Institute, 2022; Iowa Department of Human Rights & Iowa State University Extension and Outreach – Community and Economic Development [DHR & ISU Extension CED], 2024). Only immigrant-specific statistics differ in this regard. To detail each relevant group of interest: Non-English Languages speakers, English Learners in grades K–12, individuals with LEP, Spanish speakers, and Hispanics/Latinos, several tables were compiled at the state and national level (Tables 2 and 3). A separate table (Table 4) provides immigrant-specific comparisons, and all tables report two matched timepoints to illustrate relative growth over time. Compared to the typical U.S. citizen, Iowans speak fewer non-English languages, have fewer English language learners present in school, have fewer Spanish-speaking households, have fewer LEP adults, and have a smaller Latino population. However, across each category, Iowa’s percentage growth outpaces national growth, often by a factor of two or more.

**Table 2:** Relevant state averages (Iowa)

**Group of interest**	**Iowans** **(base year)**	**Base year and source**	**Iowans (recent year)**	**Recent year and source**	**Iowa rate change**
Non-English language speakers	215,629 (7.4%)	2015 U.S. Census Bureau, 2022	270,028 (8.9%)	2022 U.S. Census Bureau, 2022	+25.2% Over 7 years
English learners in grades K–12	19,580 (4%)	2010 DHR & ISU Extension CED, 2024	31,236 (6%)	2020 DHR & ISU Extension CED, 2024	+59.6% Over 10 years
Spanish language use at home	96,793 (3.2%)	2010 Migration Policy Institute, 2022	144,254 (4.8%)	2020 Migration Policy Institute, 2022	+49.0% Over 10 years
Limited English proficiency (LEP)	36,104 (1.40%)	1990 Migration Policy Institute, 2022	109,716 (3.63%)	2023 Haldar et al., 2023	+203.9% Over 23 years
Latino population	82,473 (2.8%)	2000 U.S. Census Bureau, 2020	215,986 (6.9%)	2020 U.S. Census Bureau, 2024	+161.9% Over 20 years

*Note:* Outlined are state averages for five groups of interest in Iowa: non-English language speakers, English learners in grades K–12, Spanish language use at home, individuals with limited English proficiency (LEP), and the Latino population. For each group, the table reports the raw statistic as well as its percentage relative to the state population. These values are provided for two timepoints: one recent year and one baseline year (e.g., 2010 and 2020). The percent change across those timepoints is also reported. The source and corresponding year are listed alongside each statistic.

**Table 3:** Relevant national averages (USA)

**Group of interest**	**U.S. population (recent year)**	**Recent year and source**	**U.S. rate change**
Non-English language speakers	69.2 million (22.0%)	2022 U.S. Census Bureau, 2022	+6.9% Over 7 years
English learners in grades K–12	5 million (10.1%)	2020 U.S. Department of Education NCES, 2024	+11.1% Over 10 years
Spanish language use at home	43.3 million (13.7%)	2020 U.S. Census Bureau, 2024	+23% Over 10 years
Limited English proficiency (LEP)	25.7 million (8.0%)	2022 Migration Policy Institute, 2022	+80% Over 23 years
Latino population	65.2 million (21.3%)	2020 U.S. Census Bureau, 2024	+85% Over 20 years

*Note:* Outlined are national averages for five groups of interest in the USA: non-English language speakers, English learners in grades K–12, Spanish language use at home, individuals with limited English proficiency (LEP), and the Latino population. For each group, the table reports the raw statistic as well as its percentage relative to the national population. These values are provided for a single recent year. Percent change from a prior baseline year to the recent year is also reported. The reported year and timespan match those used in Table 2, although the baseline-year national statistic is not displayed. Sources are listed alongside each reported statistic.

**Table 4:** State and national foreign-born immigrant comparisons

**Metric of comparison**	**At the state (Iowa) level**	**At the national (USA) level**
LEP foreign-born immigrants compared to:	87,063	21.11 million
(1) Total foreign-born immigrants	(1) 46.1%	(1) 46.0%
(2) Total LEP speakers in the locality	(2) 79.35%	(2) 79.69%
Hispanics/Latinos who are foreign-born immigrants compared to:	66,274	20.38 million
(1) Total foreign-born immigrants	(1) 34.7%	(1) 44.1%
(2) Total Latino population in the locality	(2) 75.19%	(2) 58.11%
Mexican immigrants compared to:	42,285	10.68 million
(1) Total foreign-born immigrants	(1) 22.17%	(1) 23.12%
(2) Total Latin American immigrants	(2) 60.18%	(2) 44.37%

*Note:* All statistics reported in the table reference data derived from 2022 estimates reported by the Migration Policy Institute and the U.S. Census Bureau (U.S. Census Bureau, 2024; Migration Policy Institute, 2022). Outlined are comparisons at both the state level (Iowa) and the national level (USA) across multiple immigrant subgroups. The table reports statistics for LEP foreign-born immigrants, Hispanics/Latinos who are foreign-born immigrants, and Mexican immigrants relative to their corresponding reference populations. For each comparison, the table reports the raw statistic as well as its percentage relative to the reference group. All statistics in the table are based on 2022 estimates from the Migration Policy Institute and the U.S. Census Bureau (U.S. Census Bureau, 2024; Migration Policy Institute, 2022).

In the case of foreign-born LEP individuals (Table 4), Iowa’s demographics closely mirror national trends. Less than a 1% difference exists between state and national proportions, suggesting that Iowa reflects a microcosm of broader immigrant LEP patterns. In both Iowa and the USA, Hispanics/Latinos constitute the largest share of LEP individuals and LEP patients seen in clinics, with most being foreign-born. Specifically, 66,274 (35.3%) of Hispanics/Latinos in Iowa are foreign-born, the majority of whom (41,688 or 67.9%) are of Mexican descent (U.S. Census Bureau, 2020). Consistent with national patterns, Mexican immigrants also represent the majority of LEP patients undergoing neuropsychological assessments in Iowa. These demographic parallels indicate that LEP is a pressing clinical concern both statewide and nationally, and that the need for equitable, linguistically appropriate neuropsychological assessment exists across all levels of care. Still, population statistics alone cannot fully explain disparities in assessment; broader systemic factors such as socioeconomic disadvantage must also be considered.

### Socioeconomic Disadvantage

Socioeconomic disadvantage can significantly compromise equitable access to neuropsychological services and healthcare at large. Nationally, >36.8 million Americans (11.1%) live in low-income, working families, and 17.23 million (5.2%) earn less than half the federal poverty threshold, adjusted for household size (Shrider & U.S. Census Bureau, 2024). Similarly, 20% of LEP individuals in the USA, an estimated 2.5 million people, live in socioeconomically disadvantaged conditions (U.S. Census Bureau, 2021). Although national assistance programs exist to help mitigate these challenges (Global Child Nutrition Foundation [GCNF], 2022), socioeconomic disadvantage can be systemic and difficult to overcome (APA, 2019; Institute of Medicine [US] Committee on Understanding and Eliminating Racial and Ethnic Disparities in Health Care 2003). Often, these disparities disproportionately affect historically disadvantaged groups, including Hispanic/Latino, immigrant, and LEP individuals.

Notably, even when scheduled, inconsistent insurance coverage, limited transportation, and competing demands from employment or caregiving may limit a patient’s ability to complete follow-up care, adhere to recommendations, or access rehabilitation services (Syed et al., 2013). Poverty can also indirectly shape neuropsychological assessment performance through its cumulative effects on education, chronic stress exposure, and healthcare inequities—all of which are known to affect neurocognitive performance (APA, 2019; Arango-Lasprilla et al., 2017). Hispanic/Latino populations are disproportionately affected by such barriers. For example, Hispanics are more likely than non-Hispanic whites to work in service, labor, and equipment operation roles, less likely to be employed in STEM fields or higher education, and more likely to live below the poverty line (16.6% compared to the national average of 11.1%) (U.S. Census Bureau, 2024). Such vulnerabilities can become especially salient in neuropsychological contexts, where test performance may reflect residual effects of sociocultural disadvantage rather than true neurocognitive dysfunction.

This concern is particularly acute for Mexican-origin patients. In a small study of Hispanic/Latina women in Michigan, U.S.-born, first-generation participants reported better health outcomes and experienced less healthcare discrimination than Mexican immigrant women (Viruell-Fuentes, 2007). Similarly, a meta-analysis comparing U.S.-born Mexican Americans and Mexican-origin immigrants found consistently higher poverty rates among immigrant families across two decades. Specifically, first-generation non-citizen children and second-generation children with two foreign-born parents experienced poverty rates of 30.2% and 25.7%, respectively, compared to ~ 17% among children with one foreign-born parent or those who were third generation or higher (Thiede & Brooks, 2018). Given these disparities, additional attention to cultural context is essential when assessing LEP immigrants. Acculturation measures can help capture some of this variability (Judd et al., 2009; Medina et al., 2023), but the selection of the scale is critical. Many commonly used tools fail to adequately reflect the nuanced cultural and linguistic experiences of Hispanic/Latino individuals, for whom generational status, migration history, and contextual exposure are important considerations (Medina et al., 2023). Without accounting for potential systemic and educational inequities, clinicians risk misinterpreting neuropsychological test scores, overpathologizing low scores, and underestimating the cognitive potential of Mexican-origin and other immigrant patients.

### The Mexican Education System Compared to the U.S. Education System

Systemic inequity in the Mexican education system is prevalent, and education in Mexico has been described as “not a basic right, but rather, a privilege of the upper classes who can afford to send their children to school” (Casas, 2010, p. 9). Although both Mexico and the USA now technically offer free public education (U.S. Congress, 2015; García et al., 2023), illegal school enrollment fees are still reported in Mexico (García et al., 2023). Key systemic differences also remain. The USA provides federally mandated supports like free or reduced-price lunch and school transportation, but these services are often absent or inconsistently implemented in Mexico and similar countries (GCNF, 2022; Ortiz-Hernández et al., 2019). For example, Mexico City only launched its first pay-to-ride school bus program in 2009 (Secretaría del Medio Ambiente del Gobierno del Distrito Federal, 2012).

Educational funding disparities further reinforce these systemic differences. In 2014, Mexico’s per-student spending was significantly below U.S. and global averages across all grade levels (Organisation for Economic Co-operation and Development [OECD], 2017, Table B1.1). Severely limited educational funding is often associated with reductions in education quality and lower levels of long-term educational and career attainment (Hopkins et al., 2007). With only 45% of individuals in Mexico completing middle school and many Mexican-origin LEP patients in the USA reporting <6 years of education (Morlett Paredes et al., 2021), educational underinvestment complicates the reliability of neuropsychological measures sensitive to educational background (Morlett Paredes et al., 2021; Suárez et al., 2020). Broader trends across Latin America, such as in Argentina, Chile, Colombia, and Costa Rica, also reflect similar educational disparities. According to the OECD (2017), all Latin American countries surveyed reported lower educational attainment (Fig. A1.2), per-student spending (Table B1.1, B1.a, and B1.2), enrollment rates (Figs. C1.1 and C1.2), and teacher salary costs (Fig. B7.1), alongside larger class sizes (Fig. D2.1) and longer teaching hours (Fig. D4.2). These data suggest systemic challenges across Latin America, despite OECD data representing only about a quarter of Spanish-speaking Latin American countries.

In contrast, roughly 90% of U.S. citizens, regardless of birthplace or school type, earn a high school diploma or GED by age 25 (Migration Policy Institute, 2022). In addition, the USA generally meets or exceeds OECD averages (OECD, 2017), which aligns more closely with the norms used in the practice of clinical neuropsychology in the USA (O’Bryant et al., 2018). Taken together, it is highly likely that the systemic issues of the Mexican and broader Latin American education systems in prior decades have limited the educational attainment of lower income Spanish-speaking immigrants who had no access to public education beyond elementary or middle school. These educational disparities may be reflected in U.S. population-level reports (i.e., U.S. census data), given that Mexican-origin individuals constitute a significant portion of Hispanic/Latino, LEP, and foreign-born individuals residing in the USA. Thus, when a U.S. speech interpreter assists an LEP patient on an interpreter-mediated neuropsychological assessment, a particular focus on educational and socioeconomic disparities, especially among Mexican-origin and other Latin American individuals, should be carefully considered during assessment.

### The Role of Educational Disparities in LEP Patient Assessment and Clinician Representation

Health services requiring specialized training, such as clinical neuropsychological assessment, are especially vulnerable to clinician–patient mismatches when working with LEP populations (DuBard & Gizlice, 2008; Elbulok-Charcape et al., 2014; Rivera Mindt et al., 2010). This is partly due to language barriers but also partly due to the limited number of multilingual clinicians and the small number of Hispanic/Latino clinical neuropsychologists nationwide (Byrd et al., 2010; Casas et al., 2012; Elbulok-Charcape et al., 2014). These workforce shortages are particularly concerning given the demographic profile of many LEP populations in the USA. For instance, Mexican-origin individuals make up the largest share of LEP individuals nationally (U.S. Census Bureau, 2020), yet educational data rarely distinguish between Hispanic/Latino subgroups, limiting insights. Consequently, educational trends are generally reported at the pan-ethnic level. Among the >65 million Hispanics/Latinos in the USA, 15.68% have less than a ninth-grade education and 26.57% have not completed high school, compared to 4.62% and 10.22% of the total U.S. population, respectively (U.S. Census Bureau, 2024b; U.S. Census Bureau., 2024a). LEP individuals show similar patterns, with 39% reporting less than a high school education (Haldar et al., 2023). This disparity is particularly consequential in neuropsychology, where educational attainment is among the strongest demographic predictors of test performance (O’Bryant et al., 2018).

Efforts to standardize and validate neuropsychological measures typically involve norming studies designed to represent a broad range of educational backgrounds (1–20 years), but individuals with the lowest levels of education are often underrepresented in these samples (Kern et al., 2008; Rodriguez-Jimenez et al., 2012; Shi et al., 2015; Suárez et al., 2020). For clinicians working with Hispanic/Latino LEP patients, many of whom report <6 years of education, this presents a major concern. In such cases, currently available co-normed batteries for U.S. Spanish-speaking adults may not provide reliable or equitable benchmarks for interpretation (Morlett Paredes et al., 2021; Suárez et al., 2020).

**Fig. 3:**
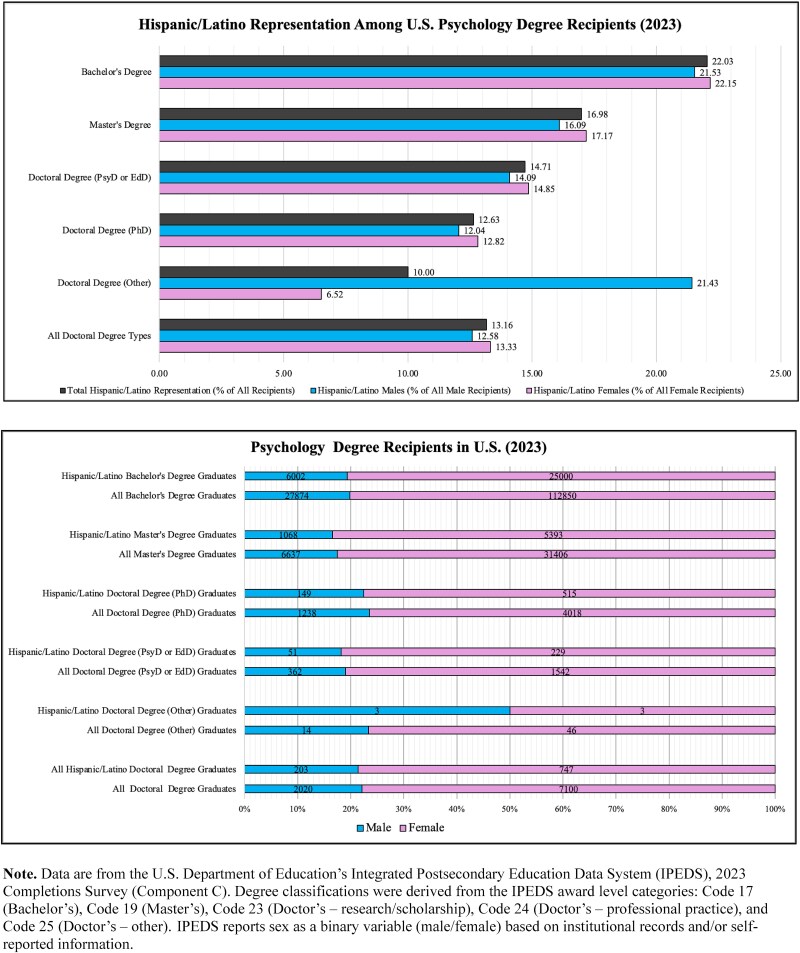
Hispanic/Latino representation among U.S. psychology degree recipients (2023). Illustrated is the representation of Hispanic/Latino students among psychology degree recipients across bachelor’s, master’s, and doctoral levels in 2023. Data are from the U.S. Department of Education’s Integrated Postsecondary Education Data System (IPEDS), 2023 Completions Survey (Component C). Degree classifications follow IPEDS award level categories: Code 17 (Bachelor’s), Code 19 (Master’s), Code 23 (Doctor’s—research/scholarship), Code 24 (Doctor’s—professional practice), and Code 25 (Doctor’s—other). Each figure consists of two graphs: the top graph shows the percentage of Hispanic/Latinos earning degrees compared to all degree earners overall, as well as percentages within male and female subgroups. The bottom graph shows the gender split at each degree classification alongside the raw counts represented by the percentages.

**Fig. 4:**
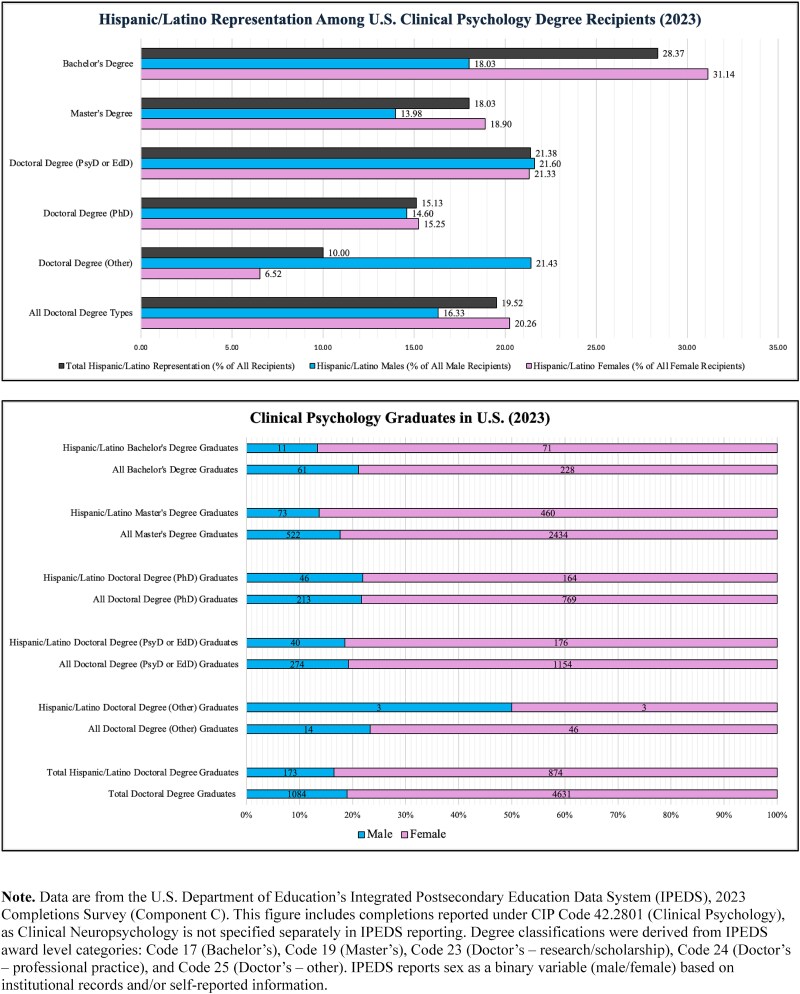
Hispanic/Latino representation among U.S. clinical psychology degree recipients (2023). Illustrated is the representation of Hispanic/Latino students among clinical psychology degree recipients in 2023. Data are from the U.S. Department of Education’s Integrated Postsecondary Education Data System (IPEDS), 2023 Completions Survey (Component C). This figure includes completions reported under CIP Code 42.2801 (Clinical Psychology), as Clinical Neuropsychology is not separately specified in IPEDS reporting. Degree classifications follow IPEDS award level categories: Code 17 (Bachelor’s), Code 19 (Master’s), Code 23 (Doctor’s—research/scholarship), Code 24 (Doctor’s—professional practice), and Code 25 (Doctor’s—other). Each figure consists of two graphs: the top graph shows the percentage of Hispanic/Latinos earning degrees compared to all degree earners overall, as well as percentages within male and female subgroups. The bottom graph shows the gender split at each degree classification alongside the raw counts represented by the percentages.

Educational disparities also affect the clinician side of neuropsychological assessment. Advanced degrees, such as those required for clinical neuropsychology, are a common marker of both career and academic achievement. However, representation in doctoral-level education remains low among historically excluded groups (Byrd et al., 2010; Elbulok-Charcape et al., 2014; Klipfel et al., 2022; Sweet et al., 2021). Due to limited reporting of clinical neuropsychology statistics across databases, clinical psychology data are referenced to contextualize clinician representation. Overall, despite representing 21.3% of the U.S. population (U.S. Census Bureau, 2022), Hispanics/Latinos comprise 12% of students enrolled across all doctoral psychology programs and 15% of students enrolled in clinical psychology programs (APA, 2024). Degree completion data is slightly less underrepresented, with Hispanics/Latinos earning 13.16% of psychology doctoral degrees (see Fig.  3) and 19.68% of clinical psychology doctoral degrees (see Fig.  4). These enrollment and degree completion metrics represent steady increases over the past decade (APA, 2024; U.S. Department of Education, National Center for Education Statistics [NCES], 2024a, 2024b) and align with the growing demand for Hispanic/Latino psychologists (IHS Markit, 2018).

Nonetheless, the growing demand for Hispanic/Latino psychologists outpaces the supply of Hispanic/Latino psychologists earning degrees (IHS Markit, 2018). Hispanics/Latinos represent only 7.78% of the general psychology workforce (APA, 2022a) and just 4.4%–4.5% of the clinical neuropsychologist workforce (Klipfel et al., 2022; Sweet et al., 2021). Notably, although only 4% of providers in the general psychology workforce report bilingual ability (APA, 2022b), 72.9% of Hispanic/Latino neuropsychologists report being Spanish–English bilinguals (Arango-Lasprilla et al., 2022). Thus, the group most suited to address the needs of Spanish-speaking LEP patients is one of the least represented in clinical neuropsychology. Additionally, many Hispanic/Latino neuropsychologists report encountering structural challenges within the field: 56.8% have reported workplace discrimination, and 57% identified inadequate mentorship as a barrier to career advancement (Arango-Lasprilla et al., 2022). These barriers not only affect long-term retention and progression of Hispanic/Latino clinical neuropsychologists but also constrain efforts to expand linguistically and culturally responsive assessment practices (Byrd et al., 2010). Considering the benefit of culturally matching clinicians (Seijo et al., 1991), the limited representation of Hispanic/Latino clinical neuropsychologists may hinder efforts to meet the needs of LEP Hispanic/Latino patients. Recognizing how these disparities shape both workforce composition and patient interactions is a necessary foundation for addressing persistent linguistic barriers and the unintended consequences of current practices.

## WHAT LINGUISTIC BARRIERS EXIST, HAVE BEEN ADDRESSED, AND HAVE ARISEN FROM THE IMPLEMENTATION OF LANGUAGE ACCOMMODATIONS IN CLINIC

### The Clinician Bottleneck

Medical context matters, so contextualizing how many clinicians are qualified to attend a patient referred for neuropsychological assessment is pertinent. At a general scale, ~14.7 million individuals are employed in a medical occupation, and across these 14.7 million employees, ~7–8 million are clinicians (U.S. Bureau of Labor Statistics, 2024). When these 7–8 million clinicians are contextualized among their respective disciplines—Physician, Registered Nurse, Nurse Practitioner, Physician Assistant, Licensed Practical Nurse, Pharmacist, Clinical Psychologist, Physical Therapist, Occupational Therapist, and Speech-Language Pathologist among other careers—7–8 million narrows to ~71,700 clinical psychologists. When only clinical neuropsychologists are considered, 71,700 narrows to roughly 5,500 clinical neuropsychologists (Sweet et al., 2021). Although these 5,500 individuals represent the broadest level of qualified clinicians able to attend a patient referred for neuropsychological assessment in the USA, not all of these individuals are actively practicing, board-certified, clinical neuropsychologists. The American Board of Professional Psychology (AACN) lists 1,167 of those individuals as actively practicing, board-certified, clinical neuropsychologists (AACN, 2024), and this represents a 99.999% reduction of the original pool of medical employees. All U.S. patients referred for neuropsychological assessment must funnel through a similar *clinician bottleneck*. A clinician bottleneck*,* a term only recently described in scientific discourse (Boes, 2024), describes the realistic limitation of patient care where the maximum quantity of patients able to be attended by a clinic cannot exceed the actual availability of the practicing clinicians. For example, in Iowa, less than 20 individuals are fully qualified to attend a patient referred for neuropsychological assessment across the entirety of the state (American Academy of Clinical Neuropsychology, 2024; American Board of Professional Psychology, 2024), and, therefore, the availability of <20 practicing clinicians bottlenecks whether or not any person in Iowa referred for neuropsychological assessment can be evaluated.

Needless to say, the demand for neuropsychological assessment for patients far exceeds clinician availability. To contextualize how disparate the clinician bottleneck is, consider the following: across 1 year in the USA, half a million individuals are diagnosed with dementia (Fang et al., 2025). Although it is preferrable for the diagnosis to come from a specialist like a clinical neuropsychologist, clinician bottlenecks severely limit the possibility of an initial diagnosis to derive from a clinical neuropsychologist and means referral sources often give the initial diagnosis for a condition. However, despite being well trained, primary care physicians (PCPs) fail to diagnose ~65% of dementia cases (Fernandes et al., 2021). In contrast, clinical neuropsychologists using formal neuropsychological assessments miss <10% of cases at initial evaluation (Schroeder et al., 2019).

#### Patient referrals and clinician language abilities

Importantly, patient referrals significantly reduce the strain of the clinician bottleneck. As referenced, referrals for neuropsychological consultation are commonly made by family physicians, neurologists, psychiatrists, and other primary care clinicians (Schroeder et al., 2019). Family physicians are the largest of these referral sources, and ~577,059 physicians are actively practicing some form of medicine today (Kaiser Family Foundation, 2024). Across these actively practicing physicians, 39.7% report speaking a language other than English (Felida et al., 2023). However, <10% of these physicians, just 22,900 physicians (3.97% of total physicians), actively utilize their multilingual skill in patient care. This, unintentionally, means that 25.70 million LEP speakers in the USA have 22,900 physicians as options rather than a potential 229,092 options. So, even across a larger pool of options at the PCP referral stage, a clinician bottleneck still exists. Notably, these estimates are also overestimates of actual availability, as not every multilingual clinician is equally available to every LEP patient. Cultural and linguistic variability, as well as whether a clinician is a linguistic match for a given patient or locally available, could significantly reduce the actual number of clinicians available. It is also important to note that these linguistic qualities apply to the referral source, not necessarily to the specialist—the clinical neuropsychologist—to whom the referral is made.

When considering specialists, it becomes apparent that the linguistic capacities of clinical neuropsychologists have yet to be described in literature (Santos et al., 2020). As a result, it remains unclear how many total clinical neuropsychologists are proficient in languages other than English or how often they utilize their multilingual skills in practice. Although, this can change. A database that lists the self-reported language abilities for active clinical psychologists exists (American Academy of Clinical Neuropsychology, 2024), but it has yet to be utilized to compile a general overview of the linguistic capacities of clinical neuropsychologists. When utilized to detail the linguistic capacities of clinical neuropsychologists in Iowa, this database indicated that all active and fully certified Iowan clinical neuropsychologists report speaking only English. However, self-reported data may underrepresent actual language capacities, and anecdotal observations from UIHC suggest this is the case. Given these limitations, the broader category of psychology health service providers can offer some additional insights. At this level, two APA reports exist (Hamp et al., 2016; APA, 2022b). Serving as a 6-year follow-up to one another, the 2015 survey reported 10.8% of respondents provided services in a non-English language, whereas the 2021 reported 10% provided them. Spanish was the most commonly provided option across both surveys (at 5.5% and 4%, respectively) and differences were nonsignificant between the reports. Overall, the reports suggest little variance in the linguistic capacities of psychologists, despite the growing need for multilingual clinicians as the number of LEP patients increases across the USA (Artiola i Fortuny & Mullaney, 1997; Judd et al., 2009; Pontón & León-Carrión, 2001; Rivera Mindt et al., 2010). In this context, speech interpreters remain a critical resource, though not without limitations that warrant further discussion.

### The Speech Interpreter Bottleneck

Similar to the clinician bottleneck, a *speech interpreter bottleneck* exists. As previously defined, a clinician bottleneck describes the realistic limitation of patient care where the maximum quantity of patients able to be attended by a clinic cannot exceed the actual availability of the practicing clinicians (Boes, 2024). Although not previously established in scientific discourse until now, the *speech interpreter bottleneck* describes the additional limitation of LEP patient care, where the maximum quantity of LEP patients able to be attended by a clinic is limited by the availability and reliability of language accessible options at that clinic. For instance, at UIHC, monolingual English clinical neuropsychologists have two options when a LEP patient arrives in clinic. If the LEP patient requests Spanish accessibility, the speech interpreter bottleneck is the sum of the two options: (1) the availability and reliability of seven medical speech interpreters on staff, and (2) the availability and reliability of the professional video-assisted speech interpretation and over-the-phone interpretation and translation services currently provided by GLOBO services (GLOBO, 2024). If an LEP patient requests a different language, such as a Chinese language—which represents the second-largest LEP language group in the USA and the third most commonly spoken language group nationally (U.S. Census Bureau, 2021; U.S. Census Bureau, 2022)—the speech interpreter bottleneck narrows to just the second option: professional video-assisted and over-the-phone interpretation services provided by GLOBO services (GLOBO, 2024).

The combination of a speech interpreter bottleneck alongside the existing clinician bottleneck can create systemic strain in clinical spaces. When systemic strains are exacerbated, public sentiments can often reflect these exacerbations. So, to contextualize whether systemic strain exists, national reporting concerning the frequency and beliefs of LEP patient visits is referenced. According to a national survey of 861 U.S. hospitals, 80% encounter LEP patients at least once a month and 63% encounter LEP patients daily or weekly (Hasnain-Wynia et al., 2006). Similarly, when 1,261 PCPs—which are a common referral group for clinical neuropsychologists—were surveyed nationally, 81% reported encountering LEP patients at least once a month with 54% encountering LEP patients daily or weekly (Ginsburg et al., 2007). Together, this indicates that LEP patients are not atypical in clinic. Additionally, those 1,261 PCPs were also asked to compare their beliefs about LEP patients to patients who speak English proficiently. The physicians overwhelmingly reported negatively on LEP patients, reporting that LEP patients were either the same as (29%) or worse (70%) at understanding basic health information, the same as (34%) or worse (64%) at asking questions to clinical staff, the same as (47%) or worse (51%) at following through on treatments, and in need of extra time often (85%). However, when the same physicians were queried about the actions they took, <10% reported engaging in any initiatives that provided or improved language services to LEP patients (Ginsburg, 2007). Whether these PCPs’ beliefs reflected reality was not clear, but their beliefs do suggest a palpable strain in clinical spaces. The extent to which this strain stems from clinician bottlenecks, speech interpreter bottlenecks, or other factors remains unclear, but the strain of the speech interpreter bottleneck can be contextualized by the availability and reliability of speech interpreters at the local, state, and national level.

Approximately 68,700 speech interpreters are currently employed in the USA (U.S. Bureau of Labor Statistics [BLS], 2024). Of the 68,700 speech interpreters in the USA, 4,584 hold an NBCMI medical interpreter license, including 25 in Iowa and 2 speech interpreters employed at UIHC (NBCMI, 2025). However, UIHC employs seven medical speech interpreters (GLOBO, 2023), raising the question of why only two of these interpreters hold NBCMI certification. The answer lies in the voluntary nature of both state and national certifications for medical interpreters. There is no legal requirement for interpreters to hold certification before practicing professionally. In fact, if you are a fluent bilingual, the successful completion of a 40-hr course is sufficient certification to work professionally as a medical interpreter for most health institutions (GLOBO, 2023). This is in stark contrast to the average 10–13 years of postsecondary education and supervised clinical neuropsychology experience required to ascertain neuropsychology licensure that is required for clinical practice (Pöchhacker & Shlesinger, 2007). As a result, the reliability of speech interpreters to retain the same degree of detail as clinical neuropsychologists is questionable, underscoring variable reliability and added strain to the speech interpreter bottleneck. For example, even when a speech interpreter can provide a technically accurate rendition of an evaluation question and a LEP patient’s response, inadequate and/or biased examinations may still occur due to a lack of a comprehensive understanding of the requirements, scoring, and/or the medical register utilized in the examination (Lezak et al., 2012). These barriers can be further exacerbated by social communication errors, such as “telephone game” effects (Mayo-Wilson, 2013) and gaps in technical training (Pöchhacker & Shlesinger, 2007). Despite these barriers, speech interpreters still play a critical role in addressing the language needs of LEP patients.

At minimum, speech interpreters are a recommendable stopgap solution. Speech interpreters serve a linguistic purpose by creating a multipurpose language-accessible tool in either SI or CI modalities in a variety of settings so that clinicians can interact with LEP patients if a language barrier is present.

Although there can be high interaction variability, professional interpreters are significantly more reliable than ad hoc interpreters. As described in prior sections, although 53% of errors among professional hospital interpreters are of clinical consequence, ad hoc interpreters show an even higher proportion at 77% (Flores et al., 2003). However, if the speech interpreter was not directly mediating the clinical encounter and was instead utilized in a limited or non-participatory manner—such as with shadow interpreters—speech interpreters might help reduce the error rates of others during assessment (Kostareva et al., 2023), though this remains to be tested empirically in clinical practice. As things stand, the ideal situation for neuropsychological assessment of LEP patients likely involves linguistically and culturally matched clinicians who routinely perform neuropsychological assessments conducting the entirety of the assessment (Casas et al., 2012; Elbulok-Charcape et al., 2014; Judd et al., 2009; Pontón & León-Carrión, 2001). This is not always a possibility. Therefore, speech interpreters should still be regarded as a useful addition to the neuropsychological assessment of LEP patients in situations where a linguistically matched clinician does not exist.

### Reported Challenges in Neuropsychological Assessment of LEP Patients

High variability in both LEP frequency and multilingual clinician availability exists across U.S. hospitals and clinics, and the related quality and accuracy of neuropsychological assessment can be highly variable from state to state, clinic to clinic, or even clinician to clinician. However, having a general understanding of the standards of practice, their improvements across time, and common issues during neuropsychological assessments in the past can help inform current practice. Currently, the delivery of culturally and linguistically appropriate neuropsychological services is reported as one of the biggest challenges in clinical neuropsychology (Casas et al., 2012), and issues related to the assessment of LEP and multilingual patients represent one of the most common ethical challenges that neuropsychologists face (Rivera Mindt et al., 2008). Hispanic/Latino LEP patients in need of neuropsychological assessments report that there are not enough bilingual neuropsychologists available to meet the demand for referrals (Casas et al., 2012). Resultantly, the American Psychological Association has consistently cited the ability to work in multiple languages as one of the most useful skills in new psychologists (Casas et al., 2012). These recommendations are made with respect to common patient concerns, as well as from concerns voiced by collaterals, surveyed before and after neuropsychological assessment. When surveyed, patients initially reported being apprehensive, but, after assessment, >90% of patients rated their experience as positive or neutral (Bennett-Levy et al., 1994). Additionally in a separate survey, patients and their collaterals reported finding neuropsychological assessments helpful in understanding and coping with cognitive problems 80% of the time, being satisfied with the evaluation more than 90% of the time, and indicated they would refer others ~90% of the time (Westervelt et al., 2007). Despite these positive survey impressions, surveys do not properly address the linguistic shortcomings historically present in neuropsychological assessment.

### Literature on the Linguistic Failings of Neuropsychological Assessment in the Past

Prior to the introduction of several standards and practices common in interpreter-mediated medical assessments in 1995, LaCalle reported that 25% of psychological and psychiatric forensic evaluations in California were conducted by a clinician who was not fluent in the patient’s primary language (La Calle, 1987). A decade later, slightly after standards and practices had been established in the USA (Avery, 1995), several exceptionally scathing critiques were penned by Lidia Artiola i Fortuny against the standards and practices of both translated and adapted assessments utilized in various neuropsychological assessments. The assessments touched on the domains of (1) Orientation and Attention, (2) Language, (3) Memory, and (4) Spatial and Praxis Abilities, and Artiola i Fortuny eviscerated the translations and adaptions of the Luria–Nebraska test battery, Mini-Mental State Examination, the “A” Cancellation Test, the Digit Symbol Test, the Spanish Naming Test, the Verbal Fluency Test, the Digit Span Test, the Memory for Unfamiliar Faces Test, the Boston Aphasia Examination, the Spanish Reading and Writing Test, the Spanish Repetition Test, the Spanish Grammar Test, the Token Test, the Calculation Abilities Test, the Verbal Serial Learning Test, the Wechsler Memory Scale Test, the Rey–Osterreith Figure, the Draw-a-Cube Test, and the Verbal Fluency Test (Artiola i Fortuny, 1996; Artiola i Fortuny & Mullaney, 1997). That same year, a national survey of neuropsychologists found that 83% of respondents did not feel adequate working with Hispanic patients and that 32% reported that their training was “totally inadequate” (Echemendia et al., 1997). The next year, Baker and colleagues published the responses of 457 emergency department patients and found that 22% of patients were unable to have access to an interpreter despite indicating a need for one (Baker et al., 1998). Two years later, although marginal improvements were incorporated into adult cognitive assessment batteries, a strong fault in pediatric age-specific assessments was detailed; specifically, the Woodcock Johnson/Woodcock Muñoz was disparaged for its inadequacies (Pontón & León-Carrión, 2001).

Things did not change in the 21st century. Two years after Ponton’s study, a systematic review found that 86% of studies evaluating quality of care reported significant negative effects of language barriers for Spanish-speaking LEP patients (Timmins, 2002). Three years later, and nearly a decade after their initial scathing publications on the majority of translated neuropsychological assessments, Artiola i Fortuny reaffirmed the unacceptable state of verbal materials and the serious threats posed by them (Artiola i Fortuny et al., 2005). Two years later, the Journal of Family Community Health characterized the state of medical interpreter-mediated assessments as “a spiraling crisis in personal and community healthcare” and listed several cultural concerns and common situations of inadvertent malpractice (Dysart-Gale, 2007). The same year, detrimental, cognate-specific effects were established in non-English translations of the Boston Naming Test (BNT) (Gollan et al., 2007). The following year, two separate meta-analyses on cross-cultural norming practices detailed the scarcity of neuropsychological measures with known procedural and interpretive equivalence across cultural groups. Pointedly, both studies concluded that the vast majority of measures utilized on neuropsychological assessment lacked proper validation for historically excluded groups, like Hispanics/Latinos (Manly, 2008; Pedraza & Mungas, 2008). Four years later, the first empirical study centered around interpreter-mediated neuropsychological assessments was realized, and it concluded “neuropsychologists are encouraged to avoid interpreter use whenever practically possible, particularly for tests with high demands on interpreter abilities and skills, with tests that have not been appropriately adapted and translated into the patient’s target language” (Casas et al., 2012). Four years later, a follow-up study of a poll done 10 years prior, in 2005, was replicated to poll U.S. and Canadian members of the International Neuropsychological Society (INS) and the National Academy of Neuropsychology (NAN) about neuropsychological assessments. The INS and NAN reported that the greatest challenges in composing the neuropsychological assessment for patients included a “lack of adequate normative data” (33.5% of respondents), “tests are culturally biased” (11.5%), and “lack of norms for additional demographic groups” (15.9%) (Rabin et al., 2016). Notably, the study stated that results were consistent with responses collected a decade prior, indicating a lack of significant improvement over a decade. However, eventually, things did begin to improve.

## ESTABLISHED RECOMMENDATIONS AND RESOLUTIONS FOR THE NEUROPSYCHOLOGICAL ASSESSMENT OF LEP PATIENTS

### Recent Improvements Addressing the Linguistic Failings of Neuropsychological Assessment

Across the last decade, despite continuing commentary on inadequacies, a shift to slightly more positive multilingual neuropsychological assessment began to be notable in the literature. In 2019, in an effort to improve the BNT and offer a Tongan version of the test, several linguistic and culturally relevant inadequacies were described to still persist for non-English norming of the BNT compared to the English standards, and recommendations were implemented in their Tongan version (Ballard et al., 2019). Two years later, a meta-analysis on norming practices across non-English adaptions of examinations highlighted the common prevalence of mismatch between the normative group and the population being evaluated and how it affected the diagnostic accuracy in cognitive disorders after neuropsychological assessment (Morlett Paredes et al., 2021). Several common instruments and their norming-relevant inadequacies were documented. In late 2024, the International Testing Commission (ITC), representing 63 countries worldwide, established guidelines for the translation and adaptation of tests used in neuropsychological assessment (Nguyen et al., 2024). The ITC formally acknowledged the widespread inadequacies and inconsistencies present in various language-specific versions of tests and issued updated recommendations, drawing on prior multinational initiatives (Franzen et al., 2021). So, although continual recommendations for improvements to assessments have gradually been proposed and implemented, issues are common and remain into the modern day (Ballard et al., 2019; Casas, 2010; Franzen et al., 2021; Judd et al., 2009; Nguyen et al., 2024; Rivera Mindt et al., 2008).

### Experimental Studies Concerning Interpreter-Mediated Neuropsychological Assessment

One study, which utilized an experimental design to quantify the extent of deficit attributable to the use of a speech interpreter on neuropsychological assessment in Puerto Rico, also exists. Although the study did not utilize clinical patients or individuals with neuropsychological concerns, the empirical study did examine triadic interpreter-mediated neuropsychological assessment in comparison to dyadic assessment using a test–retest design (Casas, 2010; Casas et al., 2012). In place of certified neuropsychologists, trained research assistants administered assessment batteries to neurologically normal research participants. These batteries were adapted from those used at the Benton Neuropsychology Laboratory at UIHC and normed by local psychometrists. Three main conclusions were drawn from the results: (1) the use of an interpreter significantly affected verbal modality scores, (2) variability in test scores generally increased for the interpreter condition, and that (3) experience in giving assessments did not have a measurable effect on scores. However, this study left the question open on whether these results would hold in a clinical setting, where patients are referred to clinic for neuropsychological assessment due to some form of neuropsychological concern.

### The Importance of Neuropsychological Assessments

Additionally, it is also important to highlight the vital role neuropsychological assessments play in addressing neuropsychological concerns. Neuropsychological assessments are essential for accurately tracking neurological changes that affect daily function, such as in cases of cognitive impairment and dementia progression (Braun et al., 2011). As a diagnostic tool, neuropsychological tests can differentiate Alzheimer’s dementia form nondementia with nearly 90% accuracy and have a 90% accuracy for diagnosing traumatic brain injury (Schroeder et al., 2019). In >50% of cases, neuropsychological testing can indicate when a patient needs assistance with daily activities, and neuropsychological assessment can serve as an evaluative metric on the potential risk of a neurologically declining individual in operating a motor vehicle (Schroeder et al., 2019). Across pediatric populations, neuropsychological assessments offer insight into developmental trajectories by identifying cognitive strengths and weaknesses, guiding educational planning, and informing diagnosis and treatment planning for conditions such as ADHD, learning disorders, and other neurodevelopmental disorders (Baron, 2018). Three primary components comprise a typical neuropsychological assessment: (1) the patient interview, where information on the patient’s personal, medical, and educational history is substantiated; (2) the test battery, where standardized neuropsychological assessments are administered to evaluate the patients cognitive, motor, and emotional function; and (3) post-assessment feedback, where findings are explained and recommendations are discussed. A neuropsychological assessment can often take 2–4 hr, depending on the patient, and an average of 12 different tests are utilized in a typical neuropsychological assessment battery; however, ~10% of clinicians do utilize >20 different tests in one encounter (Rabin et al., 2005). Neuropsychological assessments are often complementary to neuroimaging and electrophysiologic procedures, and screenings are often done to assess the appropriate examinations to be utilized. Typically, a neuropsychological assessment evaluates various domains like intellect and academic achievement, orientation and attention, memory, speech and language, visual perception and visual construction/motor construction, and mood/personality (delCacho-Tena et al., 2024). There are multiple tests to assess each cognitive domain, and every test has been normed based on a study sample of individuals across a range of age and education levels. Often, a clinical neuropsychologist takes multiple days to finalize reports, and reports are disseminated to the patients. Communications back to a referral requester, like a PCP, are also common and follow-up appointments are scheduled when sensible.

### Neuropsychological Assessment Variability in Norming

Guidelines have long existed on important areas to bracket neuropsychological assessment norming. Two of the most agreed upon areas are age and education, and both have commonly agreed upon divisions where age-specific, or education-specific, scoring factors into the final score (Lucas et al., 2005; Morlett Paredes et al., 2021; Pontón et al., 1996). Co-norming of tests—when the same sample of test takers is utilized to validate multiple tests—is also now a common standard of practice for norming neuropsychological measures (Kern et al., 2008; Rodriguez-Jimenez et al., 2012; Shi et al., 2015). Co-norming increases the clinical utility of results and the applicability of norms. Co-normed batteries that allow for comparison of performance across multiple domains of cognitive functioning among non-English languages, including Spanish, are exceptionally rare (Chang & Schallert, 2007; Morlett Paredes et al., 2021). Mismatches in norming standards across groups can lead to misdiagnosis as well (Morlett Paredes et al., 2021). For instance, when North American norms were applied to groups from Morocco, Spain, and Colombia, misdiagnosis of impairment occurred 20% of the time (Daugherty et al., 2016). In a different study specific to Hispanic/Latino LEP patients requesting Spanish, cognitive impairment was misdiagnosed 27%–31% of the time (Casaletto & Heaton, 2017). Misdiagnosis more commonly occurs when assessments evaluate patients with <6 years of education as well (Cherner et al., 2007; Morlett Paredes et al., 2021). Additionally, Western norming has a large influence on neuropsychological assessments (Rivera Mindt & Hilsabek, 2020). Even across sectors of the same country, significant variability can occur. When southwest Latinos and eastern coastal Latinos were compared in the USA, the sample norming and specifics of battery scores had regionally specific variability (Morlett Paredes et al., 2021). Overall, this underscores the importance of applying norms established on samples that resemble the population being assessed (Rivera Mindt et al., 2010; Rivera Mindt et al., 2019).

### Neuropsychological Assessment Variability across Latin America

Adapting norming to be sensitive to cultural complexity is no easy feat. Dialect, a clinician’s familiarity with an assessment, and the semantics of verbalized speech within the context of a cultural group all heavily affect neuropsychological assessment (Morlett Paredes et al., 2021; Pontón, 2001). Although the use of culturally specific norms is a notable improvement from the utilization of simple translations of assessment batteries, not all questions can be properly adapted in every test (Rosario Nieves et al., 2024). For these reasons, several neuropsychological measures have been developed in Latin America, in the Spanish language, to better suit fluent Spanish speakers abroad, including the *Batería Neuropsicológica Neuropsi, batería ECOFON, Evaluación de la Conciencia Fonológica, batería ENI*, and the *Evaluación Neuropsicológica Infantil* (Irani, 2022). Despite these advancements, the adoption of these assessments remains rare in the USA, and few hospitals have purchased copies for their clinicians. Even in Latin America, where the assessments were developed, an alarming 52% of clinical neuropsychologists report using normative data from countries outside of Latin America, and 62% report lacking normative data specific to their country (Arango-Lasprilla et al., 2017).

Overall, these factors outline some of the larger issues plaguing neuropsychological assessments catered to Hispanic/Latinos. When neuropsychologists in Latin America were surveyed, the two largest issues in order of severity were (1) the focus on the Colombian, Argentine, and Mexican population; and (2) tests being outdated (most batteries and norms were published prior to 2009) (Arango-Lasprilla et al., 2017). Ultimately, both points underscore the need for more culturally specific normative studies and neuropsychological batteries for assessments. Although adapted norms and Spanish-derived norms were consistently marked improvements from English norming, the efficaciousness of the tests has diminished with time (Irani, 2022). Thus, Spanish assessments derived in Latin America are currently notable for a low, but significant, false-positive rate. Although Spanish assessments derived in Latin America still have lower false-positive rates than when English normative data were utilized for Spanish speakers, neither the English nor Spanish normative data performed exceptionally well. In a study that evaluated neurologically normal Latin Americans (*n* = 4,866), a significant inverse correlation was established between tests administered and the probability that a cognitively normal individual scored a low score for one or more tests (Rivera et al., 2021). Across the 4,866 participants who took the five-test assessment battery, 56.4% of the sample obtained at least one low score when scored using Spanish normative data and 100% of the sample obtained at least one low score when scored using the English normative data (Rivera et al., 2021). Thus, although inroads for improvement have been made to assist LEP individuals, the current state of the field requires further improvements to the standards of neuropsychological assessment.

### Other Considerations for Neuropsychological Assessment Scoring

Although several effects that may reduce the quality and accuracy of neuropsychological assessment have been covered in prior sections, a few other notable effects exist. Two notable effects, practice effects and priming effects, detail types of learning that occur across both the short and long term that lead to overestimated cognitive abilities (Calamia et al., 2012). In order to assess cognitive rates of decline, or preservation of mind, the same individual may undergo neuropsychological assessment again in a scheduled follow-up. These individuals, if not given alternate versions of forms, may perform better upon assessment due to familiarity (whether consciously reported or not) of the examination material. Therefore, alternate forms are important to maintain evaluative validity across repeat examination. Notably, adapted versions of batteries and tests within neuropsychological assessments do not reliably offer alternative versions (Casaletto & Heaton, 2017; Chang & Schallert, 2007). Additionally, due to significant variability in how tests are normed in non-English languages (Nguyen et al., 2024), there are currently few recourses that minimize retest practice effects for returning LEP patient assessments.

Additionally, LEP patient assessments can be complicated by cognitive processes such as priming, which occurs when exposure to a particular stimulus influences an individual’s response to a subsequent prompt, often without their awareness (Schacter & Buckner, 1998). Although the priming effect is typically eliminated by modality changes between the phases of assessment, in triadic interactions—between an LEP patient, the clinician, and an interpreter—the priming effect may be significantly harder to eliminate. Additionally, when the clinician is not fluent in the language being utilized by the patient and the speech interpreter, the clinician may not even be aware priming effects are occurring during the triadic interaction. Although never empirically tested in literature, individuals who are bilingual, but not English dominant, may score higher on neuropsychological assessment due to repetition of phrases across the languages as well as tonally specific cueing that may be perceived when interpreters receive additional clarifying instruction on wording. Some evidence, in support of the effect on speech interpreters, can also be derived. As detailed previously, in cases of consecutive speech interpretation (commonly utilized in medical speech interpretation), recall about interpreter speech significantly overrepresents content from the end of the original speaker’s speech, at a statistically higher frequency than would be expected from primacy and recency effects (Dahan, 2010). Nonverbal gesturing may also prime patients during examination (Arbona et al., 2024). In triadic interactions, semantically related gestures are often performed by both the clinician and by the interpreter facilitating the patient interaction. Patients, despite linguistic limitations, can gather nonverbal information when discrete cardinal values are gestured or when iconic gesturing accompanies verbal instruction. A repeated *iconic gesture*—where the clinician gestures a semantically meaningful bit of information nonverbally and the interpreter repeats the gesture when speaking to the patient—would prime patients inadvertently to aspects of instruction (Arbona et al., 2024). Notably, in dyadic patient–clinician interactions, neither of these theoretical speech interpreter-specific priming effects would be of concern.

### Polyadic Interactions in the Clinical Interview and Post-assessment Feedback

Polyadic interactions can also exist during neuropsychological assessment. Although the administration of the test battery during neuropsychological assessment is typically either a dyadic patient–clinician interaction or a triadic patient–interpreter–clinician interaction, the patient interview prior to the test battery and the post-assessment feedback after the test battery often includes collaterals, like family members, when present. The clinical interview allows neuropsychologists to obtain informed consent; assess patient narrative reliability; confirm and collect important information about the patient’s medical history, education, employment, and upbringing; and detail a comprehensive history of the presenting complaint as well as its impact on the patient’s and family’s daily life (Casaletto & Heaton, 2017; Schaefer et al., 2018). The clinical interview includes behavioral observations and may last 1 to 2 hr. Based on the clinical interview, clinical neuropsychologists can determine the suitability of the neuropsychological battery and adjust accordingly. Additionally, it is not uncommon for clinicians to conduct collateral interviews with family members, especially in situations where the patient is potentially an unreliable narrator. After the test battery findings pertinent to the test battery are explained, recommendations are discussed with the patient and relevant collaterals, and referrals and follow-up appointments are scheduled where necessary.

Both the clinical interview and post-assessment feedback are, likely, highly affected by the use of a speech interpreter (Nielsen et al., 2024). Although literature has not yet empirically examined the effect of a speech interpreter on the clinical interview portion or the post-assessment feedback portion of a neuropsychological assessment, clinical interviews and post-assessment feedback are common medical interactions that exist outside the context of neuropsychological assessment. As such, studies derived from the fields of psychiatry and psychopathology can be utilized to derive insights about a speech interpreter’s impact on clinical interviews and post-assessment feedback because both fields have established literature on the topic. Studies from both fields overwhelmingly suggest that clinical interviews suffer from interpreter-mediated interactions. Detriments include interviewer bias arising from misaligned understandings of the medical register and misaligned cultural competencies (Cambridge, 2014; Dysart-Gale, 2007; Farooq & Fear, 2003; Jentsch, 1998), a general lack of control due to speech interpreter bottleneck–relevant effects (Cambridge, 2014; Farooq & Fear, 2003), cocktail problems from multiple speakers being present (Cooke et al., 2008; Oviatt, 2000), and reduced comprehension due to delays in receiving verbal responses (Cambridge, 2014; Farooq & Fear, 2003). During the post-assessment feedback, errors were also commonly reported with interpreter-mediated interactions. Errors made by speech interpreters included (1) omitting questions about drug allergies; (2) omitting instructions on the dose, frequency, and duration of antibiotics and rehydration fluids; (3) incorrect discernment of where to apply treatment; and (4) incorrectly restricting patient rights (Divi et al., 2007; Farooq & Fear, 2003; Flores et al., 2003). Still, significant inroads in improving the quality of interpreter-mediated neuropsychological assessment have been made over the most recent decade, and, despite significant issues still existing, the proper identification of the issues will hopefully lead to future resolutions.

## CONCLUSIONS

**Fig. 5:**
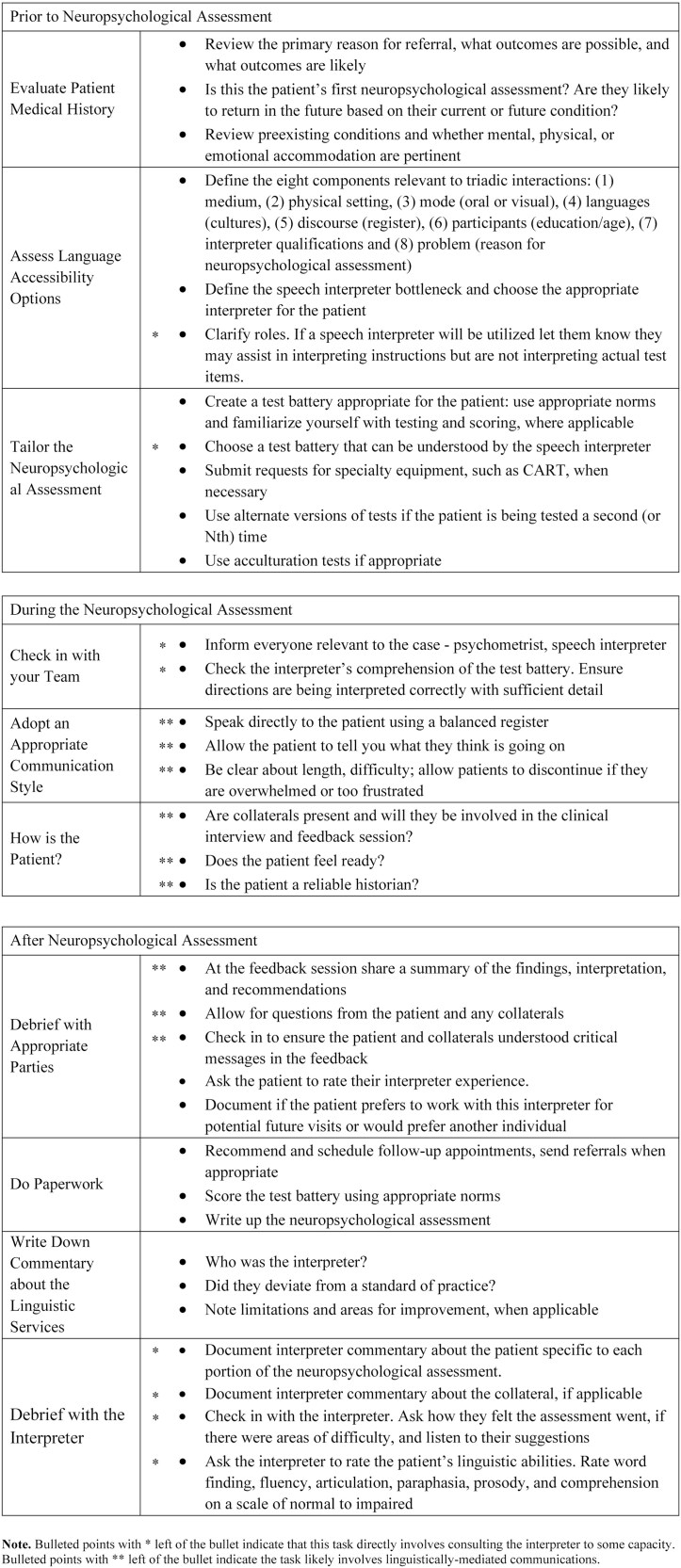
Recommendations for neuropsychological assessment of LEP patients. Illustrated are recommendations for clinical neuropsychologists at three stages of the neuropsychological assessment process: prior to assessment, during assessment, and after assessment. Bulleted points marked with a single asterisk (^*^) indicate tasks that directly involve consulting the interpreter. Bulleted points marked with a double asterisk (^**^) indicate tasks that likely involve linguistically mediated communication.

The present review expands on the current body of literature on interpreter-mediated neuropsychological assessment in the USA by detailing (1) existing language accessibility options; (2) the history of speech interpretation, clinically; (3) key distinctions in the profile of U.S. patients; (4) what linguistics barriers exist, have been addressed, and have arisen from the implementation of language accommodations in clinical practice; and (5) what recommendations or resolutions have been established for the neuropsychological assessment of LEP patients. Although many gaps exist in the field of speech interpretation and its role in neuropsychological assessment, several recommendations can be proffered. These recommendations expand on those provided in other reviews (Celik et al., 2022; Fujii et al., 2022; Nielsen et al., 2024; Santos et al., 2020) while incorporating terminology established in the field of speech interpretation (see Fig.  5).

Overall, the recommendations in this review are limited in scope and should be seen as a starting point for further exploration. Although they are designed with respect to national, state, and local variability, these recommendations specifically are structured from neuropsychological assessment practices that occurred in the USA, in the state of Iowa, and at the University of Iowa’s Hospital and Clinics primarily. Thus, Western culture, customs, and norms inevitably influence how this review has interpreted the literature and statistical reports. Notably, at the global level, additional variability exists (Fujii et al., 2022). Country-to-country variability in the availability and familiarity of clinical neuropsychologists contributes to the clinician bottleneck (Nielsen et al., 2024). The overall standards for speech interpreter training and clinical neuropsychologist licensing, as well as the availability of professional medical speech interpreters and adequate norming, also vary by country and contribute to the speech interpreter bottleneck (Nielsen et al., 2024). By incorporating linguistically focused aspects of speech interpretation into the recommendations, this review aims to encourage deeper inquiry into the field of speech interpretation and its implications in clinical neuropsychology. Gaps in our understanding of speech interpretation and neuropsychological assessment remain. Key areas for future research include the effects of interpreters on patient interviews and feedback sessions, the impact of telephonic interpretation, the impact of implementing shadow interpreters, the potential role of gesture-speak in facilitating assessments for LEP individuals, quantifying the influence of multilingualism on neuropsychological assessment (Celik et al., 2022), detailing the specific language profiles of clinical neuropsychologists in the USA and abroad (Santos et al., 2020), and quantifying the exact effect the use of a speech interpreter even has on neuropsychological assessment. With the LEP population in the USA growing, the demand for research addressing these challenges will increase, as will the potential benefits of such studies.
